# Supplementary material for: A stress-induced cilium-to-PML-NB route drives senescence initiation
Source: Nat Commun. 2023 Apr 3;14:1840. doi: 10.1038/s41467-023-37362-7 (PMC10076330; doi:10.1038/s41467-023-37362-7)
Supplement: Supplementary file 1 — Supplementary Information [file 41467_2023_37362_MOESM1_ESM.pdf]

Supplementary Materials for

A stress-induced cilium-to-PML-NB route drives senescence initiation

Xiaoyu Ma *et al*.

Correspondence to: hu.jinghua@mayo.edu

This PDF file includes:

Figs. S1 to S11

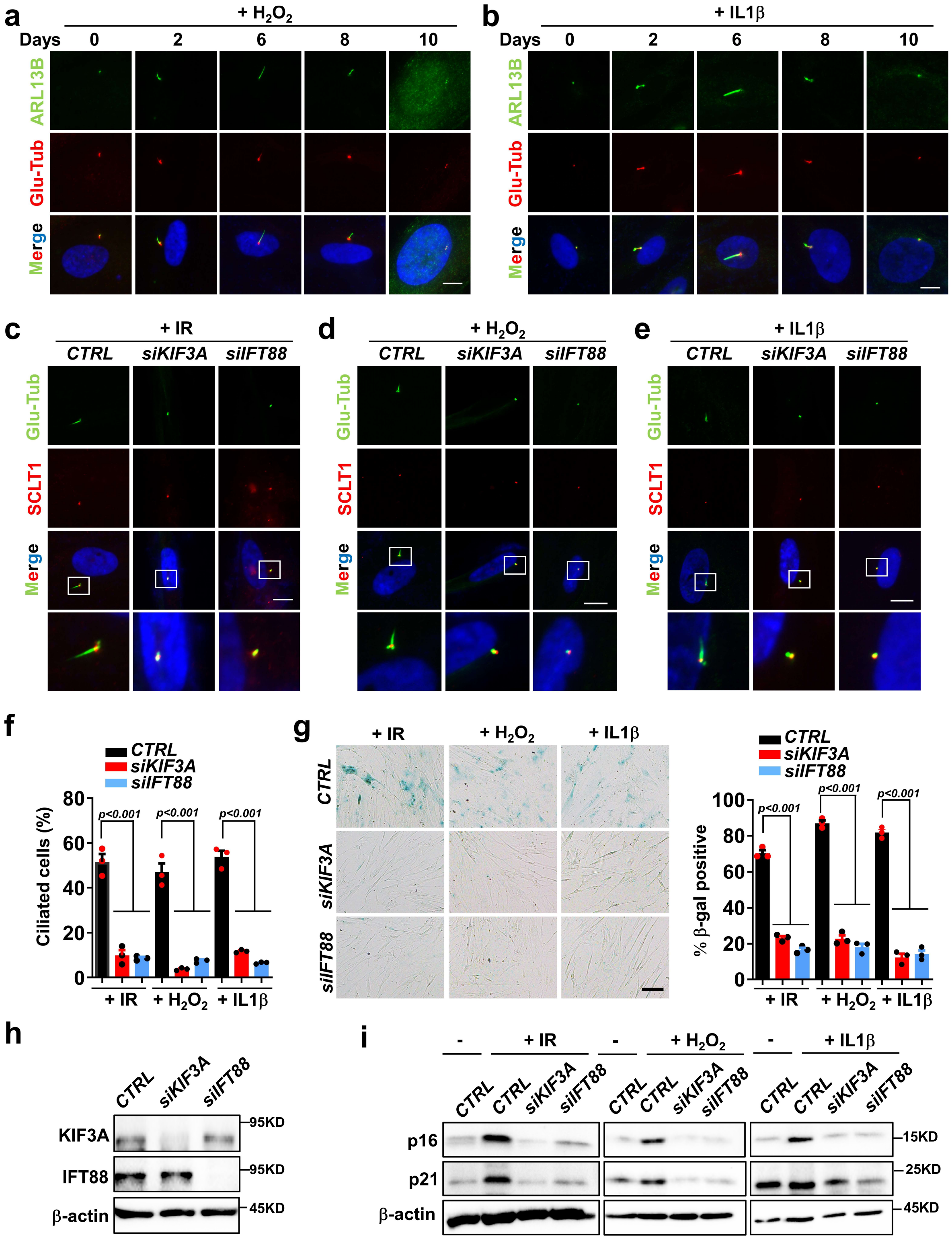

Supplementary Fig. 1. Transient ciliogenesis is required for senescence initiation.

a, Immunofluorescent staining of primary cilia in IMR-90 cells during H_2O_2-induced senescence. Primary cilia were labeled with antibodies against Glutamylated-tubulin (red) and ARL13B (green). Three experiments were repeated independently with similar results. **b,** Immunofluorescent staining of primary cilia in IMR-90 cells during IL1β-induced senescence. Cilia were labeled with antibodies against Glutamylated-tubulin and ARL13B. Three experiments were repeated independently with similar results. **c-f,** Immunofluorescent staining of primary cilia in *KIF3A* or *IFT88*-knockdown IMR-90 cells at day 2 post-irradiation (**c**), or H_2O_2 exposure (**d**), or IL1β exposure (**e**). Cilia were labeled with antibody against Glutamylated-tubulin and cilia base were labeled with antibody against SCLT1. The percentage of ciliated cells were quantified (n = 100) (**f**). **g-i,** SA-β-gal staining (n>100 cells per experiment) (**g**) and western blot of KIF3A or IFT88 (**h**) and senescence markers (**i**) in *KIF3A* or *IFT88*-knockdown IMR-90 cells at day 10 post-irradiation or at day 5 post-treatment with H_2O_2 or IL1β. Scale bar, 10 μm. Results from 3 independent experiments were statistically analyzed and plotted as means ± SEM. Two-way ANOVA followed by Bonferroni multiple-comparison analysis was used for **f** and **g**. Source data are provided as a Source Data file.

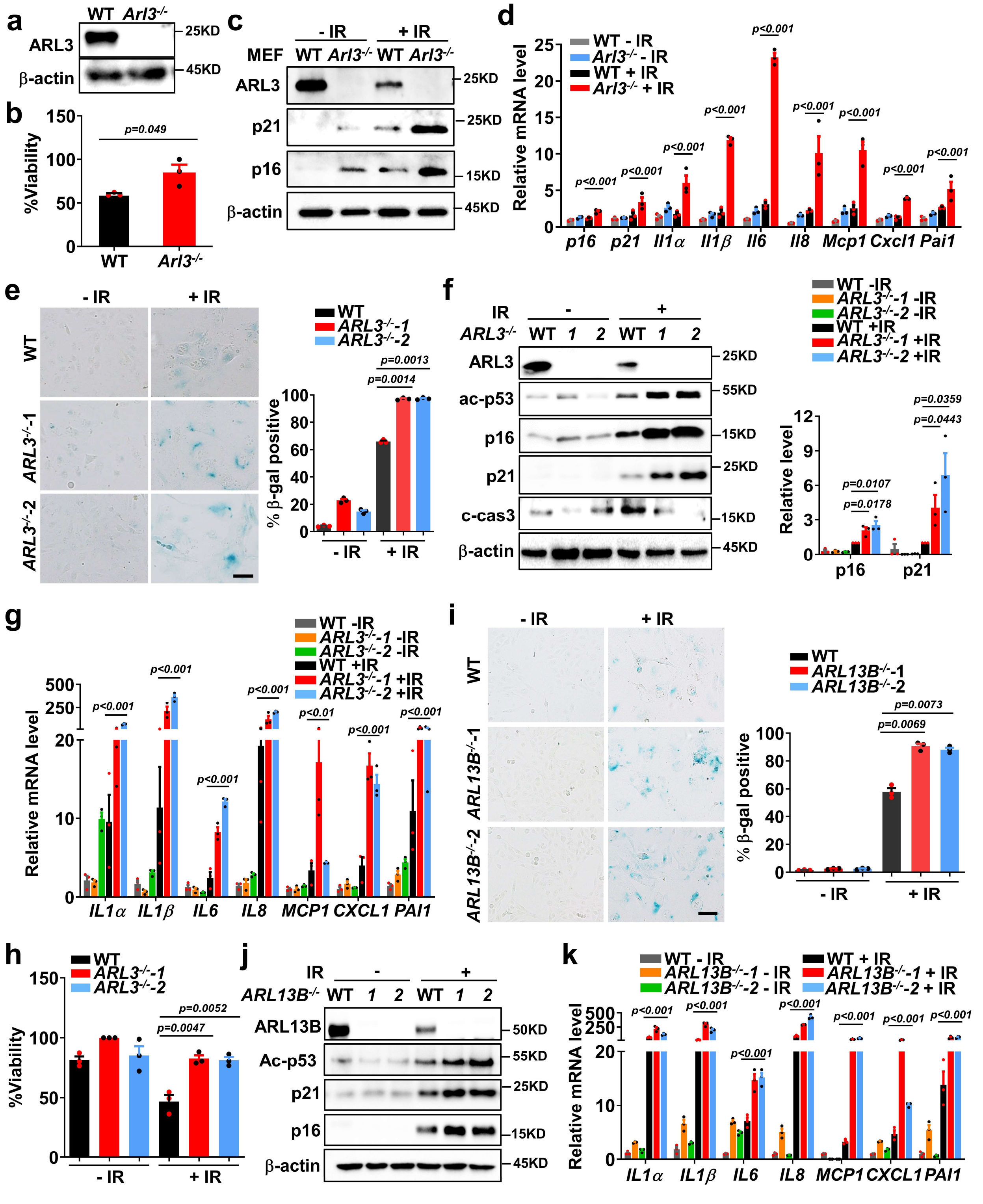

Supplementary Fig. 2. ARL3 or ARL13B deficiency promotes IR-induced senescence. a, Western blot of ARL3 in WT, *Arl3*$^{-/-}$ MEF cells. **b,** Viability assay of WT or *Arl3*$^{-/-}$ MEF cells with

IR exposure. **c, d,** Western blot of senescence markers (**c**) and relative mRNA levels of SASP genes (**d**) in WT or *Arl3*$^{-/-}$ MEF cells without or with IR exposure at day 7 post-irradiation. **e-g,** SA-β-gal staining (n>100 cells per experiment) (**e**), western blot of senescence markers (**f**) and relative mRNA levels of SASP genes (**g**) in WT or *ARL3*$^{-/-}$ RCTE cells at day 7 post-irradiation. **h,** Viability assay of WT or *ARL3*$^{-/-}$ RCTE cells without or with IR exposure at day 7 post-irradiation. **i-k,** SA-β-gal staining (n>100 cells per experiment) (**i**), western blot of senescence markers (**j**) and relative mRNA levels of SASP markers (**k**) in WT or *ARL13B*$^{-/-}$ RCTE cells at day 7 after irradiation. Scale bar, 200 μm. Results (**b, d-h, k-h**) from n=3 independent experiments were statistically analyzed and plotted as means ± SEM. Two-tailed Student's unpaired t-test was used for analysis in **b, f** and **h**. Two-way ANOVA followed by Bonferroni multiple-comparison analysis was used for **d, g** and **k**. Brown-Forsythe and Welch ANOVA tests was used for **e** and **i**.

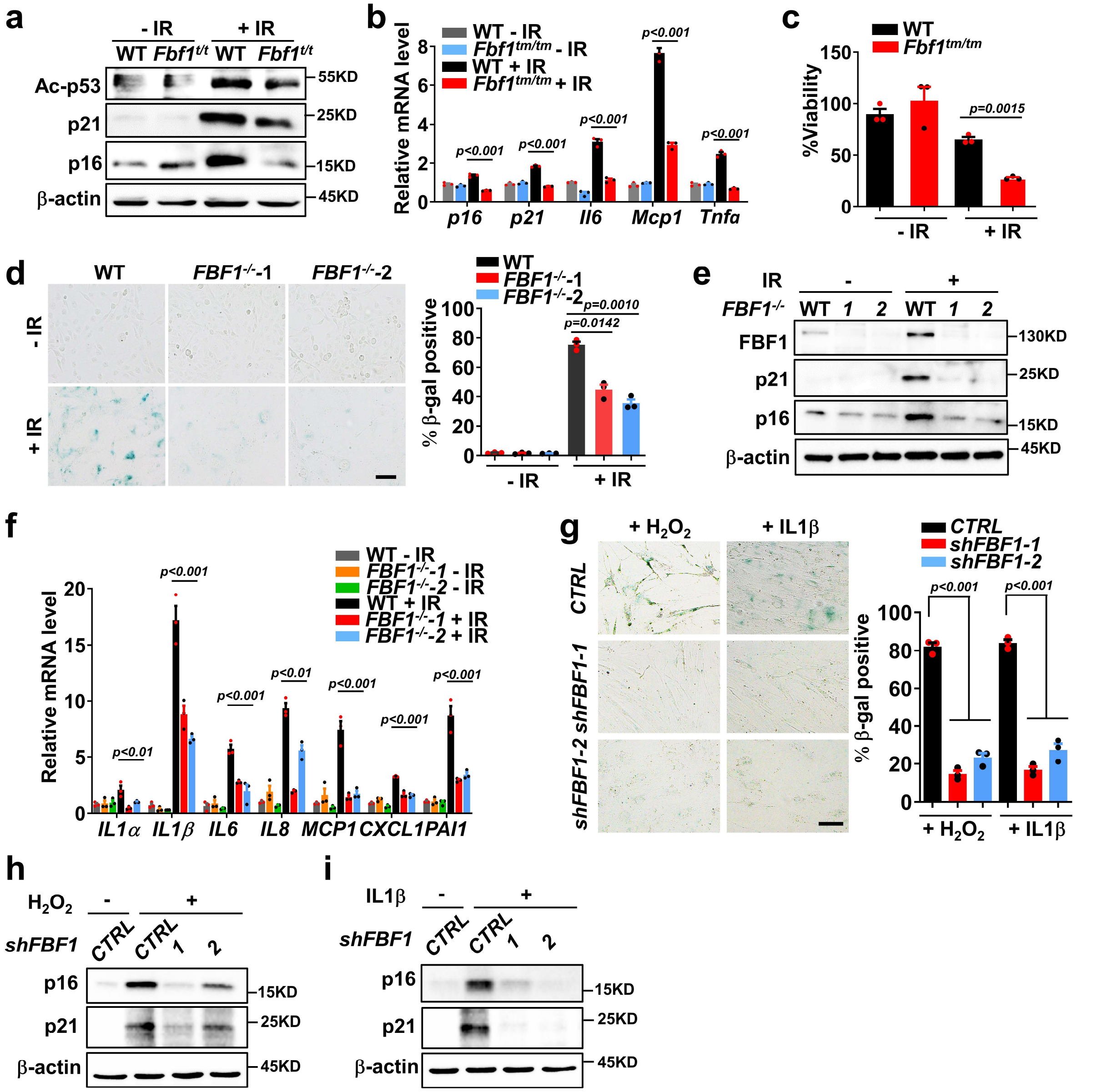

Supplementary Fig. 3. FBF1 deficiency suppresses senescence induction. a-c, Western blot of senescence markers (**a**), relative mRNA levels of SASP genes (**b**), and viability assay (**c**) in WT or *Fbf1*$^{tm1a/tm1a}$ MEF cells without or with IR exposure at day 7 post-irradiation. **d-f,** SA-β-gal staining (n>100 cells per experiment) (**d**), western blot of senescence markers (**e**) and relative mRNA levels of SASP genes (**f**) in WT or *FBF1*$^{-/-}$ RCTE cells at day 7 after irradiation. Scale bar, 200 μm. **g,** SA-β-gal staining in control or sh*FBF1* IMR-90 cells at day 5 post H_2O_2 or IL1β

treatment (n>100 cells per experiment). **h-i,** western blot of senescence markers in control or sh*FBF1* IMR-90 cells at day 5 post H_2O_2 (**h**) or IL1β treatment (**i**). Three experiments were repeated independently with similar results. Results (**b-d, f-g**) from n=3 independent experiments were statistically analyzed and plotted as means ± SEM. Two-way ANOVA followed by Bonferroni multiple-comparison analysis was used for **b** and **f**. Two-tailed Student's unpaired t-test was used for analysis in **c**. Brown-Forsythe and Welch ANOVA tests was used for **d** and **g**.

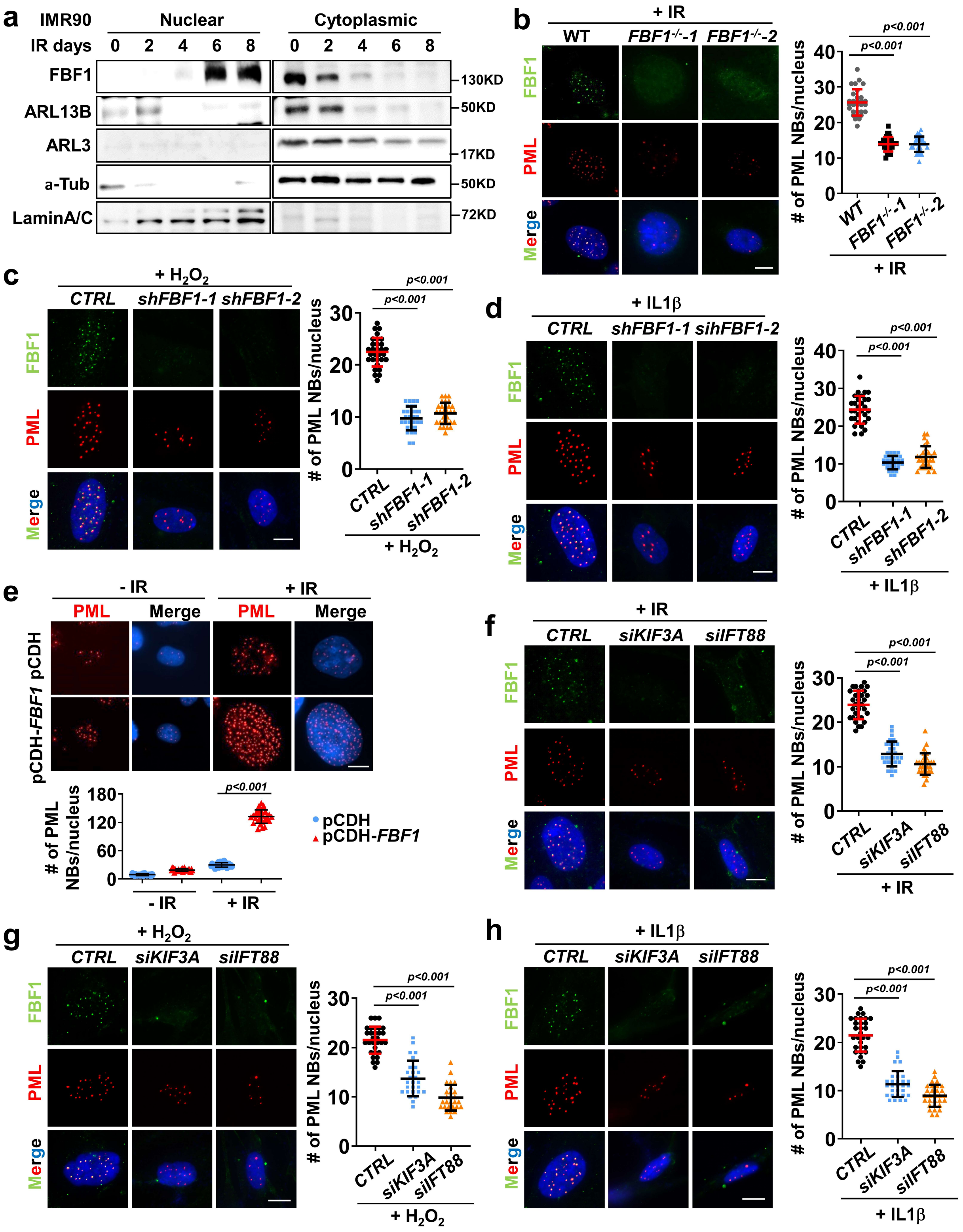

Supplementary Fig. 4. Primary cilia are required for IR-induced PML-NB translocation of FBF1 and PML-NB biogenesis. a, Western blot of ARL3, ARL13B and FBF1 in

nuclear and cytoplasmic fractions separated from IMR-90 cells after irradiation at indicated times. Three experiments were repeated independently with similar results. **b,** Immunofluorescent images for FBF1 and PML in WT or *FBF1*$^{-/-}$ RCTE cells at day 7 post-irradiation. n=30 cells. Scale bar, 10 µm. **c-d,** localization of FBF1 and PML in H_2O_2 (**c**) or IL1β (**d**) treated control or *FBF1*-knockdown IMR-90 cells. n=30 cells. Scale bar, 10 µm. **e,** PML staining in RCTE cells overexpressed with plasmid pCDH vector or pCDH-FBF1-Myc at day 7 after irradiation. n=30 cells. Scale bar, 10 µm. **f,** Immunofluorescent images for FBF1 and PML in *KIF3A* or *IFT88*-knockdown IMR-90 cells at day 10 after irradiation. n=30 cells. Scale bar, 10 µm. **g-h**, localization of FBF1 and PML in *KIF3A* or *IFT88*-knockdown IMR-90 cells at day 5 post H_2O_2 (**g**) or IL1β (**h**) treatment. n=30 cells. Scale bar, 10 µm. Results (**b-h**) from n=3 independent experiments were statistically analyzed and plotted as means ± SEM. One-way ANOVA followed by Bonferroni multiple-comparison analysis was employed for **b-h.**

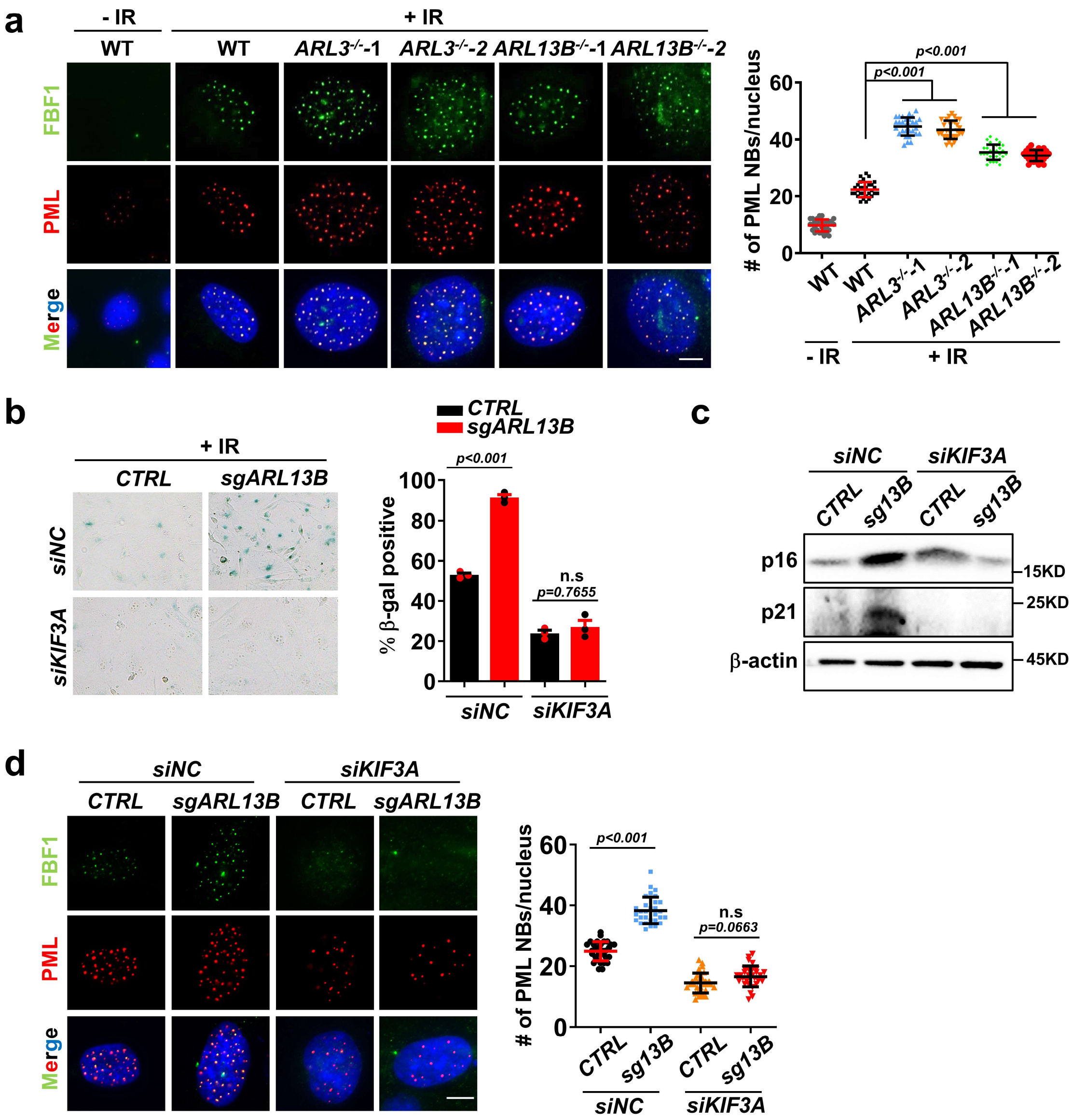

Supplementary Fig. 5. Cilia suppression abrogates ARL13B-defeiciency-induced senescence responses. **a**, Immunofluorescent images for FBF1 and PML in WT or *ARL3*$^{-/-}$ or *ARL13B*$^{-/-}$ RCTE cells at day 7 after irradiation. n=30 cells. Scale bar, 10 µm. **b-d,** SA-β-gal staining (n>100 cells per experiment) (**b**), western blot of senescence markers (**c**) and localization of FBF1 and PML (**d**) in si*KIF3A*-treated WT or *ARL13B*$^{-/-}$ RCTE cells at day 7 post-irradiation. n=30 cells. Scale bar, 10 µm. Results (**a-b, d**) from n=3 independent experiments were statistically analyzed and plotted as means ± SEM. One-way ANOVA followed by Bonferroni multiple-comparison analysis was employed for **a** and **d**. Brown-Forsythe and Welch ANOVA tests was used for **b**.

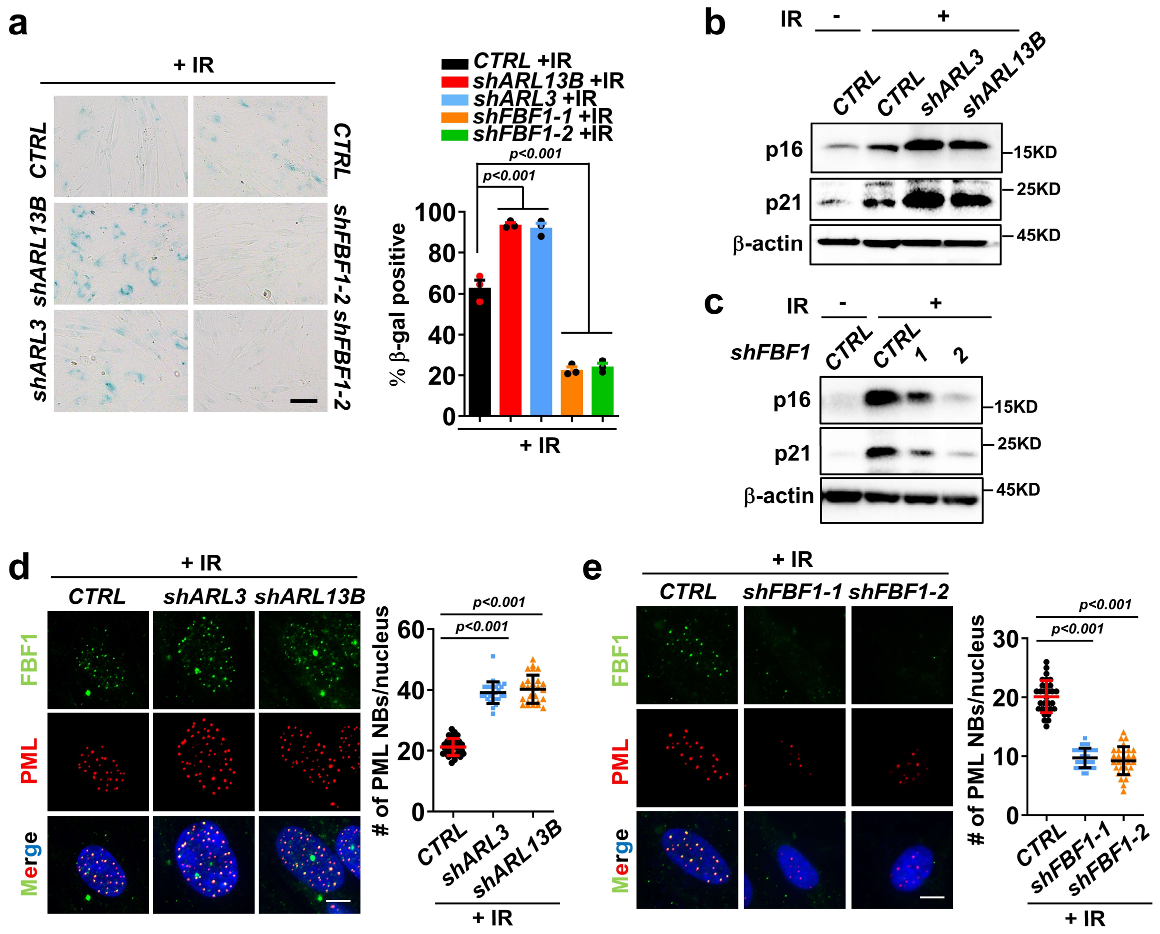

Supplementary Fig. 6. ARL13B, ARL3 or FBF1 are key players in IR-induced senescence in BJ cells. a, SA-β-gal staining in BJ cells stably expressing sh*ARL13B or* sh*ARL3* or sh*FBF1* at day 10 after irradiation (n>100 cells per experiment). **b-c,** western blot of senescence markers in *ARL3 or ARL13B*-knockdown (**b**) or *FBF1*-knockdown (**c**) BJ cells at day 10 post-irradiation. Three experiments were repeated independently with similar results. **d-e,** localization of FBF1 and PML in *ARL3 or ARL13B*-knockdown (**d**) or *FBF1*-knockdown (**e**) BJ cells at day 10 post-irradiation. n=30 cells. Scale bar, 10 µm. Results (**a, d-e**) from n=3 independent experiments were statistically analyzed and plotted as means ± SEM. One-way ANOVA followed by Bonferroni multiple-comparison analysis was employed for **a, d** and **e**.

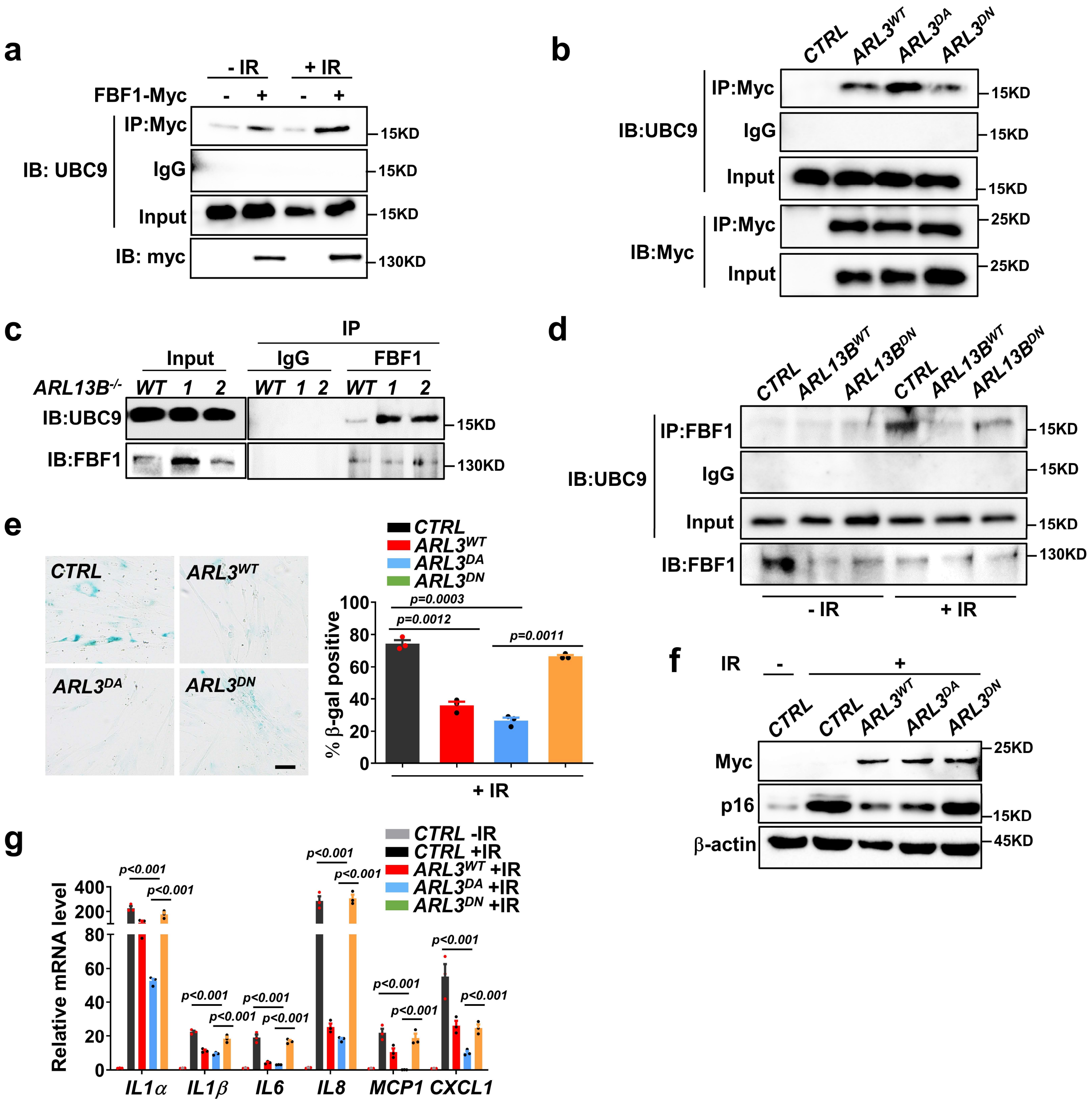

Supplementary Fig. 7. The ARL13B-ARL3 GTPase cascade suppresses FBF1 SUMOylation and cellular senescence. a, Myc-tagged FBF1 immunoprecipitates with endogenous UBC9 in RCTE cells overexpressing FBF1 without or with IR at day 7 post treatment. **b,** Anti-Myc antibody immunoprecipitates with endogenous UBC9 in RCTE cells overexpressing Myc-tagged ARL3^{WT}, ARL3^{DA} or ARL3^{DN} at day 7 after IR treatment. **c,** Endogenous FBF1 immunoprecipitates with UBC9 in WT or *ARL13B*$^{-/-}$ RCTE cells at day 7 post-irradiation. **d,** Endogenous FBF1 immunoprecipitates with UBC9 in RCTE cells overexpressing ARL13B^{WT} or ARL13B^{DN} at day 7

post-irradiation. **e-g,** SA-β-gal staining (n>100 cells per experiment) (**e**), western blot of senescence markers (**f**), and relative mRNA levels of SASP genes (**g**) in IMR-90 cells overexpressing $ARL3^{WT}$, $ARL3^{DA}$ or $ARL3^{DN}$ at day 10 post-irradiation. Scale bar, 200 μm. Three experiments were repeated independently with similar results (**a-d**). Results (**e, g**) from n=3 independent experiments were statistically analyzed and plotted as means ± SEM. Brown-Forsythe and Welch ANOVA tests was used for **e**. Two-way ANOVA followed by Bonferroni multiple-comparison analysis was used for **g**.

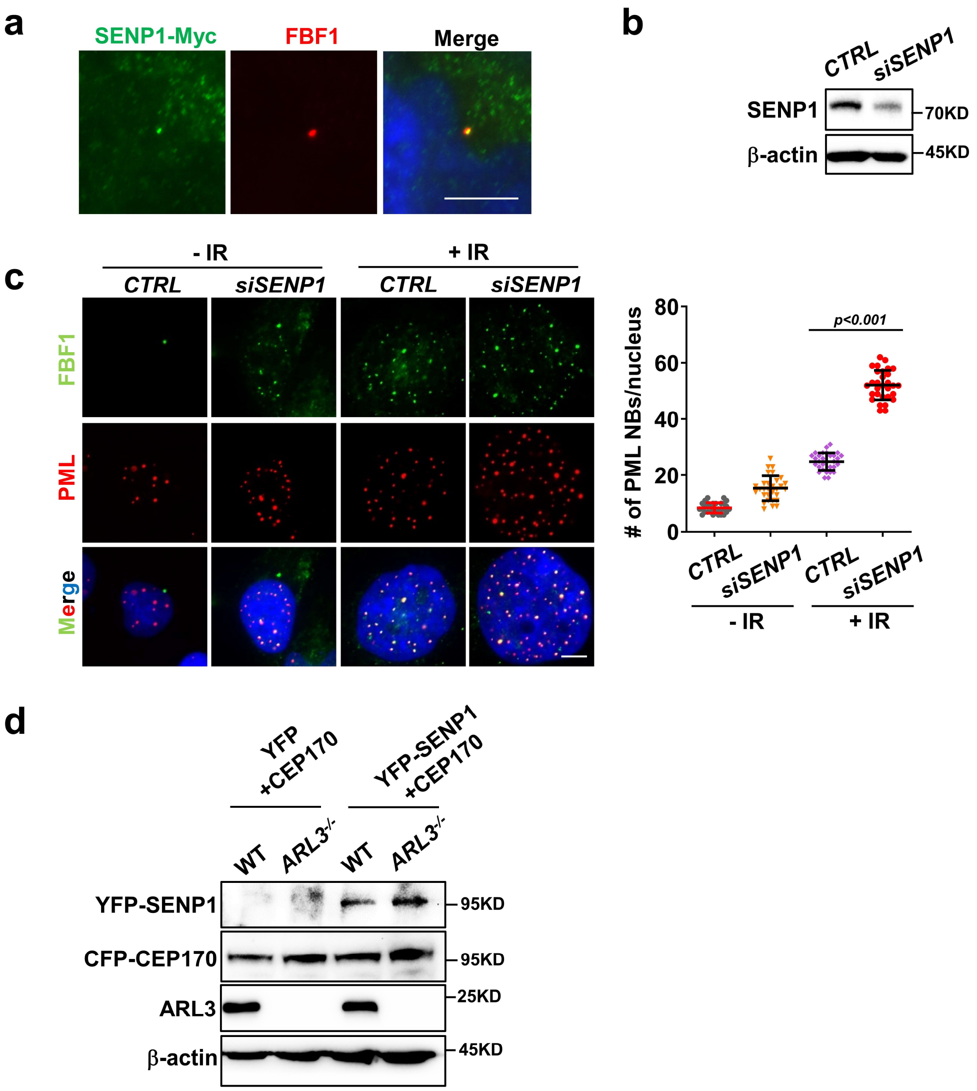

Supplementary Fig. 8. SENP1 directly regulates FBF1 SUMOylation and its PML-NB translocation. a, Immunofluorescent images for Myc-tagged SENP1 and FBF1 staining in RCTE cells. Scale bar, 10 μm. Three experiments were repeated independently with similar results. **b,** Western blot of SENP1 in control or *SENP1*-knockdown RCTE cells. Three experiments were repeated independently with similar results. **c,** Immunofluorescent images for FBF1 and PML staining in control or *SENP1*-knockdown RCTE cells without or with IR at day 7 post-irradiation. n=30 cells. Scale bar, 10 μm. **d,** Western blot of WT or *ARL3*$^{-/-}$ RCTE cells co-transfected with CEP170c-CFP-FRB and YFP-FKBP-SENP1. Three experiments were repeated independently with similar results. Results (**c**) from n=3 independent experiments were statistically analyzed and

plotted as means ± SEM. One-way ANOVA followed by Bonferroni multiple-comparison analysis was employed for **c**.

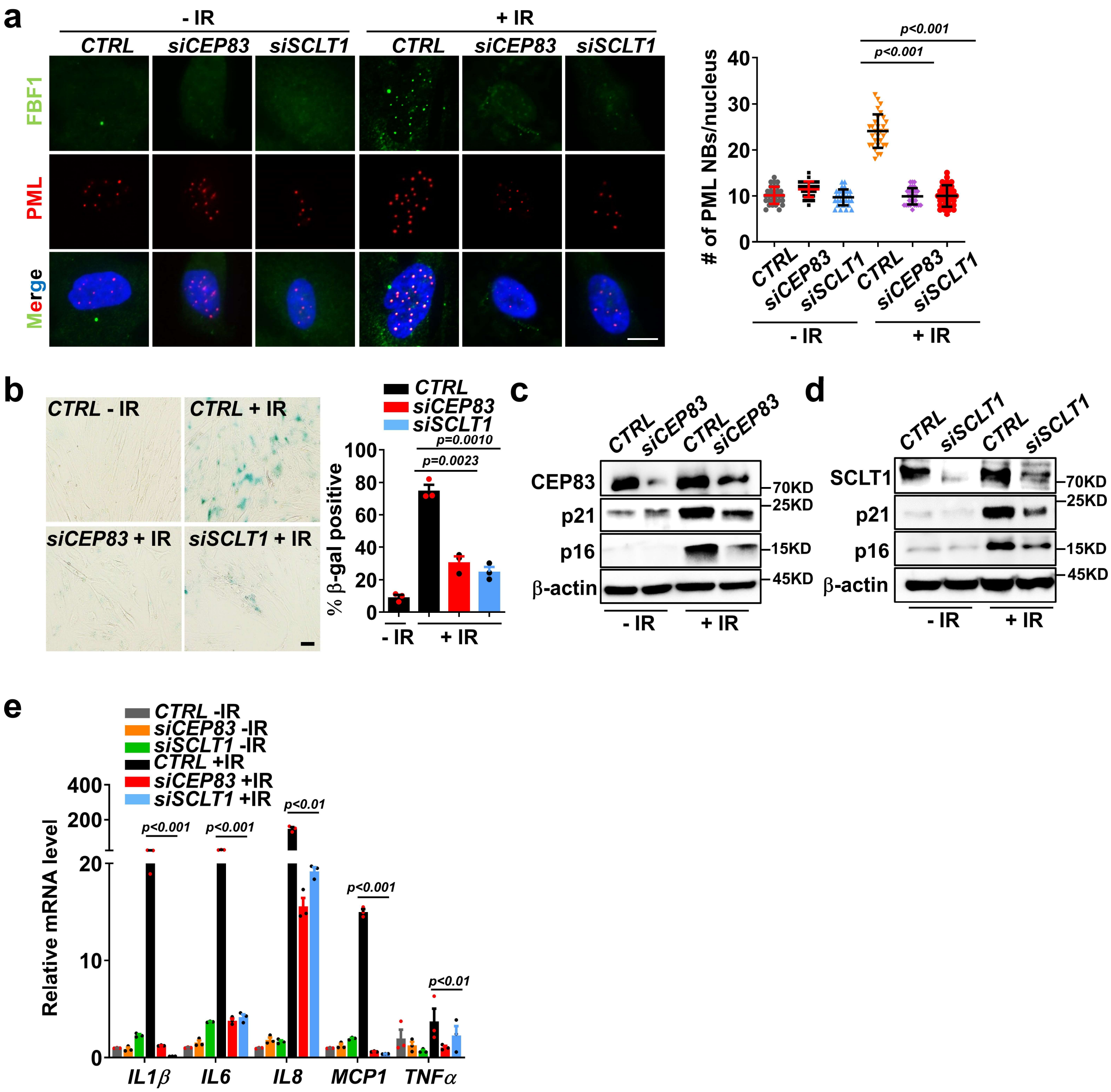

Supplementary Fig. 9. TF localization of FBF1 is a prerequisite for IR-induced PML-NB translocation and senescence initiation in IMR-90 cells. a, Immunofluorescent images for FBF1 and PML staining in control, *CEP83*-knockdown, or *SCLT1*-knockdown IMR-90 cells without or with IR at day 7 post-irradiation. n=30 cells. Scale bar, 10 μm. **b-e,** SA-β-gal staining (n>100 cells per experiment) (**b**), western blot of senescence markers (**c, d**) and relative mRNA levels of SASP genes (**e**) in control, *CEP83*-knockdown, or *SCLT1*-konckdown IMR-90 cells at day 7 post-irradiation. Scale bar, 20 μm. Results (**a-b, e**) from n=3 independent experiments were statistically

analyzed and plotted as means ± SEM. One-way ANOVA followed by Bonferroni multiple-comparison analysis was employed for **a**. Brown-Forsythe and Welch ANOVA tests was used for **b**. Two-way ANOVA followed by Bonferroni multiple-comparison analysis was used for **e**.

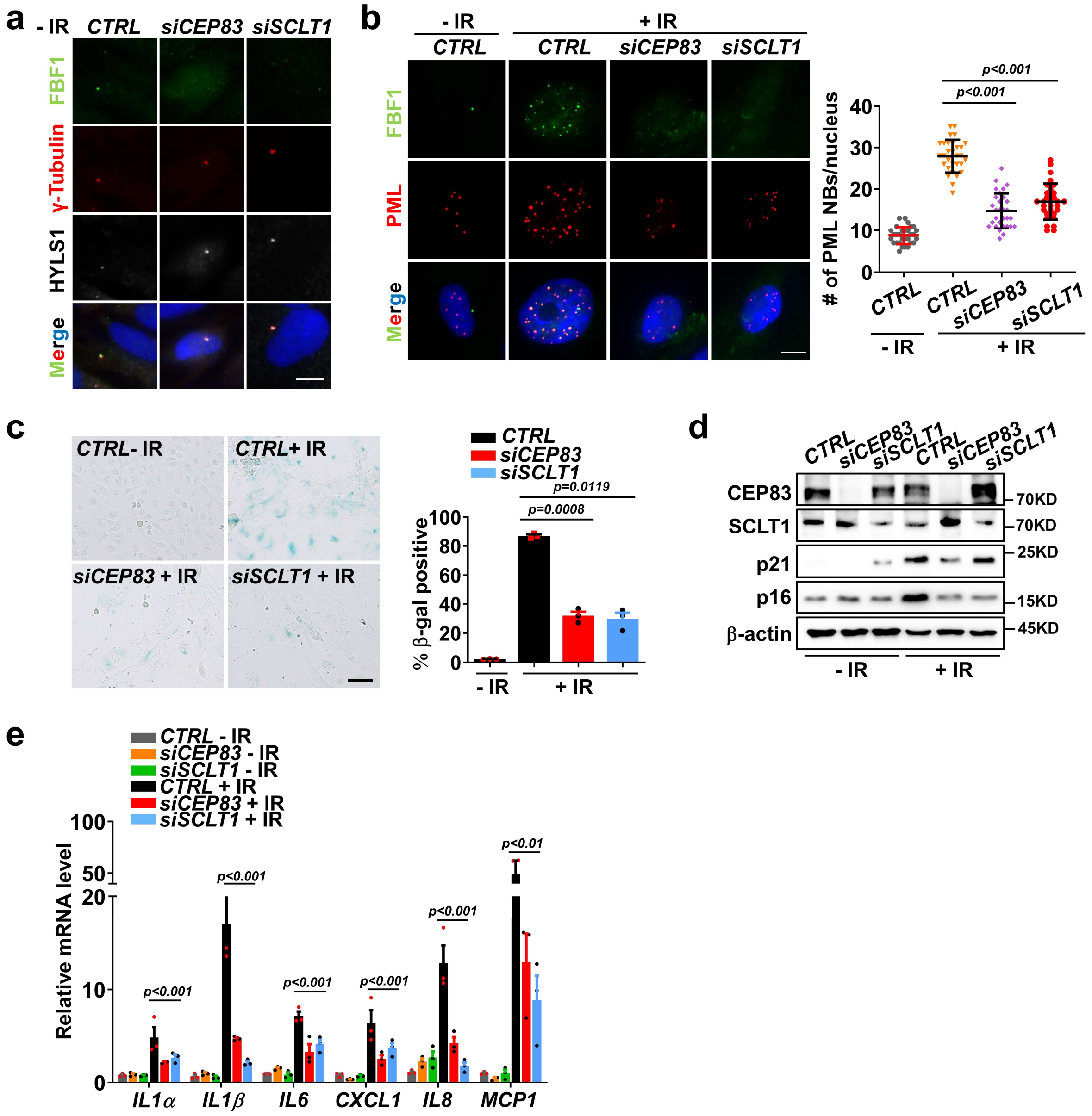

Supplementary Fig. 10. TF localization of FBF1 is a prerequisite for its role in senescence initiation in RCTE cells. a, b, Immunofluorescent images for FBF1, γ-tubulin and HYLS1 (**a**) or PML (**b**) in control or *CEP83*-knockdown or *SCLT1*-knockdown RCTE cells without or with IR at day 7 post-irradiation. n=30 cells. Scale bar, 10 μm. **c-e,** SA-β-gal staining (n>100 cells per experiment) (**c**), western blot of senescence markers (**d**) and relative mRNA levels of SASP markers (**e**) in control, *CEP83*-knockdown, or *SCLT1*-konckdown RCTE cells at day 7 post-irradiation. Scale bar, 200 μm. Results (**b, c, e**) from n=3 independent experiments were

statistically analyzed and plotted as means ± SEM. One-way ANOVA followed by Bonferroni multiple-comparison analysis was employed for **b**. Brown-Forsythe and Welch ANOVA tests was used for **c**. Two-way ANOVA followed by Bonferroni multiple-comparison analysis was used for **e**.

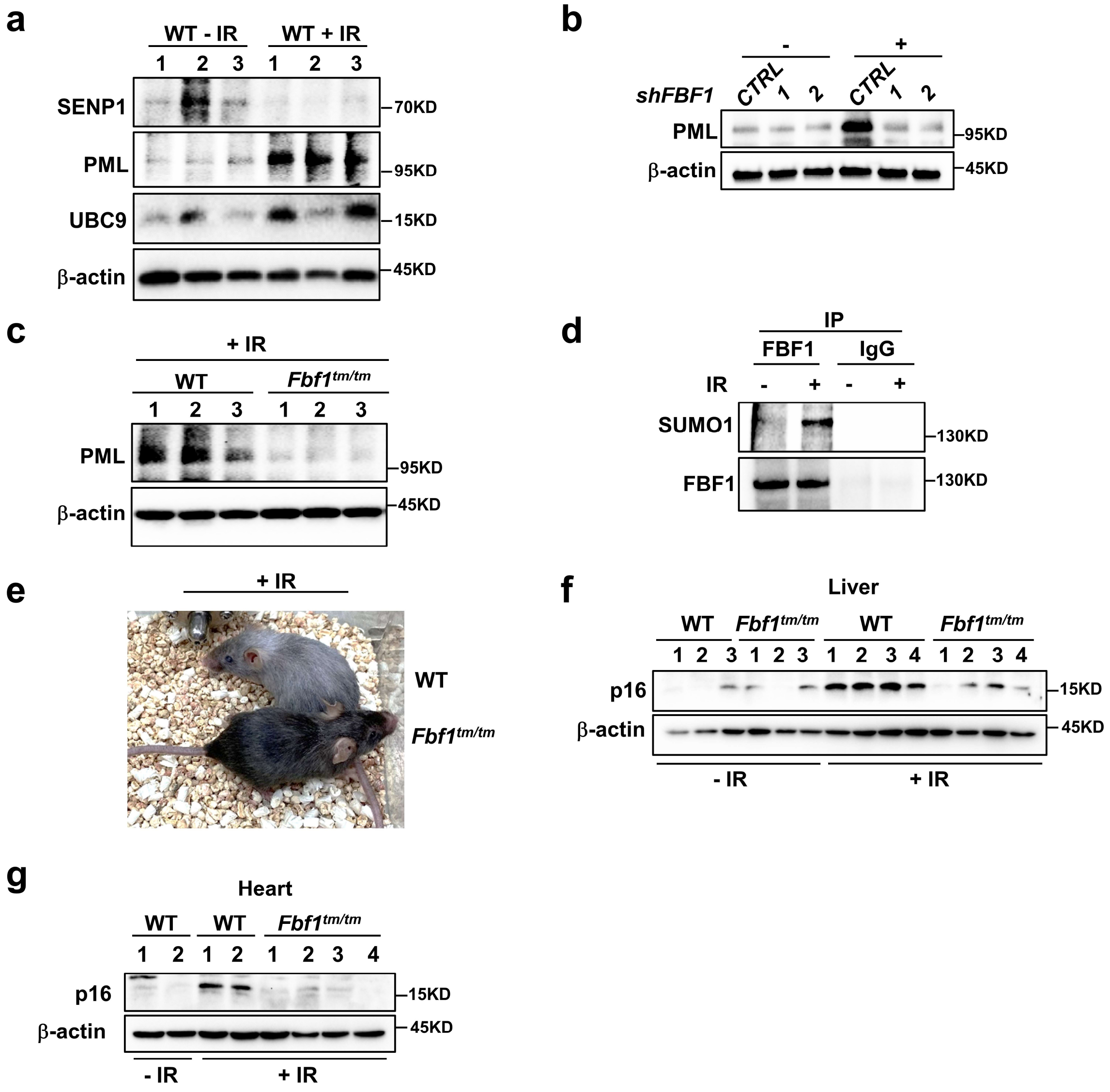

Supplementary Fig. 11. FBF1 ablation protects mice from IR-induced senescence and associated frailty. a, Lysates obtained from lung tissue of WT mice two weeks post IR were subjected to western blot analysis against indicated proteins. **b**, Western blot of PML in control or sh*FBF1* IMR-90 cells at day 10 post-irradiation. **c,** protein level of PML in lung tissue of WT mice or *Fbf1*$^{tm1a/tm1a}$ mice two weeks post IR. **d,** Endogenous FBF1 immunoprecipitates with SUMO1 in lung tissue of WT mice with or without irradiation. **e,** Representative images of mice one year post IR. **f-g,** Lysates obtained from liver (**f**) and heart (**g**) tissue of WT or *Fbf1*$^{tm1a/tm1a}$ mice two

weeks post IR were subjected to western blot analysis against indicated proteins. Three experiments were repeated independently with similar results (**a-d, f-g**).
