# Supplementary material for: A stress-induced cilium-to-PML-NB route drives senescence initiation
Source: Nat Commun. 2023 Apr 3;14:1840. doi: 10.1038/s41467-023-37362-7 (PMC10076330; doi:10.1038/s41467-023-37362-7)
Supplement: Supplementary file 3 — Description of Additional Supplementary Files [file 41467_2023_37362_MOESM3_ESM.pdf]

File name: Supplementary Data 1

Description: Sequences of oligonucleotides.
