# Supplementary material for: A stress-induced cilium-to-PML-NB route drives senescence initiation
Source: Nat Commun. 2023 Apr 3;14:1840. doi: 10.1038/s41467-023-37362-7 (PMC10076330; doi:10.1038/s41467-023-37362-7)
Supplement: Supplementary file 5 — Reporting Summary [file 41467_2023_37362_MOESM5_ESM.pdf]

nature portfolio

Corresponding author(s): Jinghua Hu
Last updated by author(s): 2/23/2023

Reporting Summary

Nature Portfolio wishes to improve the reproducibility of the work that we publish. This form provides structure for consistency and transparency in reporting. For further information on Nature Portfolio policies, see our Editorial Policies and the Editorial Policy Checklist.

Statistics

For all statistical analyses, confirm that the following items are present in the figure legend, table legend, main text, or Methods section.

n/a	Confirmed	
☐	☒	The exact sample size (*n*) for each experimental group/condition, given as a discrete number and unit of measurement
☐	☒	A statement on whether measurements were taken from distinct samples or whether the same sample was measured repeatedly
☐	☒	The statistical test(s) used AND whether they are one- or two-sided<br>*Only common tests should be described solely by name; describe more complex techniques in the Methods section.*
☒	☐	A description of all covariates tested
☐	☒	A description of any assumptions or corrections, such as tests of normality and adjustment for multiple comparisons
☐	☒	A full description of the statistical parameters including central tendency (e.g. means) or other basic estimates (e.g. regression coefficient) AND variation (e.g. standard deviation) or associated estimates of uncertainty (e.g. confidence intervals)
☐	☒	For null hypothesis testing, the test statistic (e.g. *F*, *t*, *r*) with confidence intervals, effect sizes, degrees of freedom and *P* value noted<br>*Give P values as exact values whenever suitable.*
☒	☐	For Bayesian analysis, information on the choice of priors and Markov chain Monte Carlo settings
☒	☐	For hierarchical and complex designs, identification of the appropriate level for tests and full reporting of outcomes
☒	☐	Estimates of effect sizes (e.g. Cohen's *d*, Pearson's *r*), indicating how they were calculated

Our web collection on statistics for biologists contains articles on many of the points above.

Software and code

Policy information about availability of computer code

Data collection	ZEN - ZEISS Efficient Navigation, Nikon Elements (NIS ElementsAR ver. 4.6.0.) were used to acquire Immunofluorescence images.<br>ChemiDoc™ Touch Imaging System were used to acquire western blot images.<br>CFX384 Real-Time PCR Detection Systems were used to acquire qRT-PCR results.<br>All-in-one microplate reader software Gen5 2.07 were used to acquire MTT assay results.
Data analysis	- Immunofluorescence images were contrast, overlay and analyzed with NIS ElementsAR ver. 4.6.0.<br>- Western blot images were contrast and analyzed with Image Lab (Bio-Rad)and ImageJ.<br>- Real-Time PCR results were analyzed with CFX manager (Bio-Rad).<br>- Statistical analyses: All data were analyzed with GraphPad Prism (version 5 and 9) and Microsoft Excel.

For manuscripts utilizing custom algorithms or software that are central to the research but not yet described in published literature, software must be made available to editors and reviewers. We strongly encourage code deposition in a community repository (e.g. GitHub). See the Nature Portfolio guidelines for submitting code & software for further information.

Data

Policy information about availability of data

All manuscripts must include a data availability statement. This statement should provide the following information, where applicable:

- Accession codes, unique identifiers, or web links for publicly available datasets
- A description of any restrictions on data availability
- For clinical datasets or third party data, please ensure that the statement adheres to our policy

No dataset were generated in this study. All materails are available from the corresponding author upon reasonable request.

Human research participants

Policy information about studies involving human research participants and Sex and Gender in Research.

Reporting on sex and gender	N/A
Population characteristics	N/A
Recruitment	N/A
Ethics oversight	N/A

Note that full information on the approval of the study protocol must also be provided in the manuscript.

Field-specific reporting

Please select the one below that is the best fit for your research. If you are not sure, read the appropriate sections before making your selection.

☒ Life sciences ☐ Behavioural & social sciences ☐ Ecological, evolutionary & environmental sciences

For a reference copy of the document with all sections, see nature.com/documents/nr-reporting-summary-flat.pdf

Life sciences study design

All studies must disclose on these points even when the disclosure is negative.

Sample size	Sample size was determined to be adequate based on the magnitude and consistency of measurable differences between groups. It was also chosen on the basis of prior studies that showed significant effects with similar sample sizes. For experiments involving quantification of PML numbers in cells, sample size was determined by the number of cells within the replicates and based on similar studies in this field. The minimal animal size used in previously published literature is 5 per gender (Winer DA et al, Nature, 2011).
Data exclusions	Data were not excluded from analysis.
Replication	In this study, all attempts at replication were successful. Each result described in the paper is based on at least three independent biological replicates but very often an experiment is based on more than three experiments. Figure legends indicate the number of independent experiments performed in each analysis.
Randomization	For animal experiments, mice of both genders were randomly allocated to experimental and control groups. For other experiments, samples were randomly allocated to experimental and control groups.
Blinding	For animal experiments, the investigators were not blinded for genotyping. This is because blinding requires mixing lines of different genotypes (mutant and wt lines), which could lead to a risk of mislabeling. For all other cell based experiments, the investigators were blinded in data analysis.

Reporting for specific materials, systems and methods

We require information from authors about some types of materials, experimental systems and methods used in many studies. Here, indicate whether each material, system or method listed is relevant to your study. If you are not sure if a list item applies to your research, read the appropriate section before selecting a response.

Materials & experimental systems

n/a	Involved in the study	
☐	☒	Antibodies
☐	☒	Eukaryotic cell lines
☒	☐	Palaeontology and archaeology
☐	☒	Animals and other organisms
☒	☐	Clinical data
☒	☐	Dual use research of concern

Methods

n/a	Involved in the study	
☒	☐	ChIP-seq
☒	☐	Flow cytometry
☒	☐	MRI-based neuroimaging

Antibodies

Antibodies used

Primary antibodies:
1. Rabbit polyclonal anti-ARL13B, Proteintech, Cat#17711-1-AP; Lot#00077350; dilution 1:2000 for immunofluorescent and 1:1000 for western blotting.
2. Rabbit polyclonal anti-FBF1, Proteintech, Cat#11531-1-AP; Lot#00039337; dilution 1:1000 for immunofluorescent and western blotting.
3. Rabbit polyclonal anti-ARL3, Proteintech, Cat#10961-1-AP; Lot#00040401; dilution 1:1000 for immunofluorescent and western blotting.
4. Rabbit polyclonal anti-p16INK4a, Proteintech, Cat#10883-1-AP; Lot#00095293; dilution 1:1000 for western blotting.
5. Rabbit polyclonal anti-CEP83, Proteintech, Cat#26013-1-AP; Lot#00025954; dilution 1:1000 for immunofluorescent and western blotting.
6. Rabbit polyclonal anti-SCLT1, Proteintech, Cat#14875-1-AP; Lot#00005967; dilution 1:1000 for immunofluorescent and western blotting.
7. Mouse monoclonal anti-IL6, Proteintech, Cat#66146-1-Ig; Lot#10003220; dilution 1:1000 for western blotting.
8. Rabbit polyclonal anti-IL1a, Proteintech, Cat#16765-1-AP; Lot#00008153; dilution 1:200 for immunohistochemistry.
9. Rabbit polyclonal anti-KIF3A, Proteintech, Cat#13930-1-AP; Lot#00049919; dilution 1:1000 for western blotting.
10. Rabbit polyclonal anti-IFT88, Proteintech, Cat#13967-1-AP; Lot#00010704; dilution 1:1000 for western blotting.
11. Rabbit polyclonal anti-SUMO-1 (human) (NT), Enzo Life Sciences, Cat#BML-PW8330-0025; Lot#01031927; dilution 1:1000 for western blotting.
12. Mouse monoclonal anti-SUMO1 (D-11), Santa Cruz, Cat#sc-5308, Lot#J2511; dilution 1:500 for western blotting.
13. Mouse monoclonal anti-UBC9 (C-12), Santa Cruz, Cat#sc-271057, Lot#A2119; dilution 1:500 for western blotting.
14. Mouse monoclonal anti-p16INK4a, Abcam, Cat#ab54210; dilution 1:200 for immunohistochemistry.
15. Rabbit polyclonal anti-p16INK4a, StressMarq Biosciences, Cat#SPC-775D; Lot#PC786014; dilution 1:500 for western blotting.
16. Mouse monoclonal anti-acetylated tubulin clone 6-11B-1, Sigma, Cat#T7451, Lot#0000132783; dilution 1:5000 for immunofluorescent.
17. Mouse monoclonal anti-glutamylated tubulin (GT335), AdipoGen Life Science, Cat# AG-20B-0020-C100; dilution 1:1000 for immunofluorescent.
18. Mouse monoclonal anti-β-actin, clone AC-15, Sigma, Cat#A1978; dilution 1:2000 for western blotting.
19. Mouse monoclonal anti-p21, Santa Cruz, Cat#sc-6246, Lot#L2921; dilution 1:500 for western blotting.
20. Mouse monoclonal anti-PML (PG-M3), Santa Cruz, Cat#sc-966, Lot#G2018; dilution 1:500 for immunofluorescent.
21. Mouse monoclonal anti-PML (E-11), Santa Cruz, Cat#sc-377390, Lot#B1119; dilution 1:200 for western blotting.
22. Mouse monoclonal anti-SENP1 (C-12), Santa Cruz, Cat#sc-271360, Lot#K0817; dilution 1:500 for immunofluorescent and western blotting.
23. Mouse monoclonal anti-HYLS1 (D-9), Santa Cruz, Cat#sc-376721, Lot#K0719; dilution 1:200 for immunofluorescent.
24. Rabbit polyclonal anti-ac-p53, Cell Signaling technology, Cat#2525, Lot#16; dilution 1:1000 for western blotting.
25. Mouse monoclonal anti-FLAG tag, Sigma, Cat#F1804; dilution 1:2000 for western blotting.
26. Mouse monoclonal anti-HA tag, clone HA-7, Sigma, Cat#H3663; dilution 1:2000 for western blotting.
27. Mouse monoclonal anti-Myc tag, clone GT0002, Sigma, Cat#SAB2702192; dilution 1:2000 for western blotting.
28. Mouse monoclonal anti-GST tag (B-14), Santa Cruz, Cat#sc-138, Lot#G2318; dilution 1:1000 for western blotting.
29. Rabbit polyclonal anti-His tag, Proteintech, Cat#66005-1-Ig; Lot#10004365; dilution 1:1000 for western blotting.

Secondary antibodies:
1. Peroxidase-AffiniPure Goat anti-mouse, Jackson ImmunoResearch Laboratories, Cat# 111-035-144; dilution 1:2000 for western blotting.
2. Peroxidase-AffiniPure Goat anti-rabbit, Jackson ImmunoResearch Laboratories, Cat# 115-035-146; dilution 1:2000 for western blotting.
3. Goat anti-Rabbit IgG (H+L) Highly Cross-Adsorbed Secondary Antibody, Alexa Fluor 488, Invitrogen, Cat# A-11034; dilution 1:1000 for immunofluorescent.
4.Goat anti-Rabbit IgG (H+L) Highly Cross-Adsorbed Secondary Antibody, Alexa Fluor 555, Invitrogen, Cat#A-21429; dilution 1:1000 for immunofluorescent.
5.Goat anti-Mouse IgG1 Cross-Adsorbed Secondary Antibody, Alexa Fluor 555, Invitrogen, Cat# A-21127; dilution 1:1000 for immunofluorescent.
6. Goat anti-Mouse IgG1 Cross-Adsorbed Secondary Antibody, Alexa Fluor 488, Invitrogen, Cat#A-21121; dilution 1:1000 for immunofluorescent.
7. Goat anti-Mouse IgG2a Cross-Adsorbed Secondary Antibody, Alexa Fluor 647, Invitrogen, Cat#A-21241; dilution 1:1000 for immunofluorescent.
8. Goat anti-Mouse IgG1 Cross-Adsorbed Secondary Antibody, Alexa Fluor 647, Invitrogen, Cat#A-21240; dilution 1:1000 for immunofluorescent.
9. Goat anti-Mouse IgG2b Cross-Adsorbed Secondary Antibody, Alexa Fluor 647, Invitrogen, Cat#A-21242; dilution 1:1000 for immunofluorescent.

Validation	1. https://www.ptglab.com/products/ARL13B-Antibody-17711-1-AP.htm<br>2. https://www.ptglab.com/products/FBF1-Antibody-11531-1-AP.htm<br>3. https://www.ptglab.com/products/ARL3-Antibody-10961-1-AP.htm<br>4. https://www.ptglab.com/products/P16,P19-Antibody-10883-1-AP.htm<br>5. https://www.ptglab.com/products/CCDC41-Antibody-26013-1-AP.htm<br>6. https://www.ptglab.com/products/SCLT1-Antibody-14875-1-AP.htm<br>7. https://www.ptglab.com/products/IL6-Antibody-66146-1-Ig.htm<br>8. https://www.ptglab.com/products/IL1A-Antibody-16765-1-AP.htm<br>9. https://www.ptglab.com/products/KIF3A-Antibody-13930-1-AP.htm<br>10. https://www.ptglab.com/products/IFT88-Antibody-13967-1-AP.htm<br>11. https://www.enzolifesciences.com/BML-PW8330/sumo-1-human-nt-polyclonal-antibody/<br>12. https://www.scbt.com/p/sumo-1-antibody-d-11<br>13. https://www.scbt.com/p/ubc9-antibody-c-12<br>14. https://www.abcam.com/cdkn2ap16ink4a-antibody-2d9a12-ab54210.html<br>15. https://www.stressmarq.com/products/antibodies/cdkn2a-antibody-spc-775/?v=7516fd43adaa<br>16. https://www.sigmaaldrich.com/US/en/product/sigma/t7451<br>17. https://adipogen.com/ag-20b-0020-anti-polyglutamylation-modification-mab-gt335.html/<br>18.https://www.sigmaaldrich.com/US/en/product/sigma/a1978?<br>gclid=CjwKCAiA9NGfBhBvEiwAq5vSy35OdybBn9kSj97xFSQcNGM7ey44kmWAd71WOBY5KRzlS0MvNlI1SBoC2iEQAvD_BwE&gclsrc=aw<br>.ds<br>19. https://www.scbt.com/p/p21-antibody-f-5<br>20.https://www.scbt.com/p/pml-antibody-pg m3?<br>gclid=CjwKCAiA9NGfBhBvEiwAq5vSyw1flAeaUwXJl2PTBnVbseGc29ewx7U0Qs1vSjH-wZWL-vyeuEO1uRoCYU4QAvD_BwE<br>21. https://www.scbt.com/p/pml-antibody-e-11<br>22. https://www.scbt.com/p/senp1-antibody-c-12<br>23. https://www.scbt.com/p/hyls1-antibody-d-9<br>24. https://www.cellsignal.com/products/primary-antibodies/acetyl-p53-lys382-antibody/2525<br>25.https://www.sigmaaldrich.com/US/en/product/sigma/f1804?<br>gclid=CjwKCAiA9NGfBhBvEiwAq5vSy9d78lsLyVgjacTeXHsa9V0NH2hL56Sx8CZaSzZa6oRhWb3LbZe8vxoCYUkQAvD_BwE&gclsrc=aw.ds<br>26. https://www.sigmaaldrich.com/US/en/product/sigma/h3663?gclid=CjwKCAiA9NGfBhBvEiwAq5vSy-<br>fV6NuUaVZjbEgwnd4NEn0i9Q11XjoPISlsLA6GBX9sT0UEwoBMBxoCNjgQAvD_BwE&gclsrc=aw.ds<br>27. https://www.sigmaaldrich.com/US/en/product/sigma/sab2702192<br>28. https://www.scbt.com/p/gst-antibody-b-14<br>29. https://www.ptglab.com/products/His-Tag-Antibody-66005-1-Ig.htm

Eukaryotic cell lines

Policy information about cell lines and Sex and Gender in Research

Cell line source(s)	IMR90 (human, ATCC Cat# CCL-186); BJ (human, ATCC Cat# CRL-2522); RCTE (human, ATCC Cat# CRL-4031); 293T (human, ATCC Cat# CRL-3216)
Authentication	Cell identities were confirmed routinely by western blot and immunoflourescence marker expressions.
Mycoplasma contamination	Cell lines were routinely tested for mycoplasma contamination by PCR and confirmed that they were negative for mycoplasma contamination.
Commonly misidentified lines<br>(See ICLAC register)	No commonly misidentified cell lines were used.

Animals and other research organisms

Policy information about studies involving animals; ARRIVE guidelines recommended for reporting animal research, and Sex and Gender in Research

Laboratory animals	C57BL/6J mice were purchased from the Jackson Laboratory. The Fbf1tm1a/tm1a mice were obtained from Trust Sanger Institute and refreshed the genetic background as described (Zhang et al., 2021). The Arl3 floxed mouse line was a gift from Dr. Wolfgang Baehr's lab (Hanke-Gogokhia et al., 2016). They are all C57BL/6J background. Both females and males, 6 months of age, were used in this study.Mice were maintained in a pathogen-free facility at 23–24 °C with relative humidity no higher than 50 percent under a 12 h light-dark cycle regimen with free access to a NCD (standard mouse diet Lab Diet 5053, St. Louis, MO) and water. Mice were bred and housed under a Specific Pathogen-Free (SPF) status. All mice were housed in static autoclaved HEPA-ventilated microisolator cages (27 × 16.5 × 15.5 cm) with autoclaved Enrich-o'Cobs (The Andersons Incorporated) for bedding. Cages and bedding were changed biweekly. Cages were opened only in class II biosafety cabinets. The majority of subjects from the study are the result of in-house mating, and littermate controls were used.
Wild animals	No wild animals were used in this study.
Reporting on sex	Mice of both genders were randomly allocated to different groups.
Field-collected samples	No field-collected samples were used in this study.

Ethics oversight | All animal experiments were performed according to protocols approved by the Institutional Animal Care and Use Committee (IACUC) at the Mayo Clinic.

Note that full information on the approval of the study protocol must also be provided in the manuscript.
