# Supplementary figures and images for: A stress-induced cilium-to-PML-NB route drives senescence initiation
Source: Nat Commun. 2023 Apr 3;14:1840. doi: 10.1038/s41467-023-37362-7 (PMC10076330; doi:10.1038/s41467-023-37362-7)

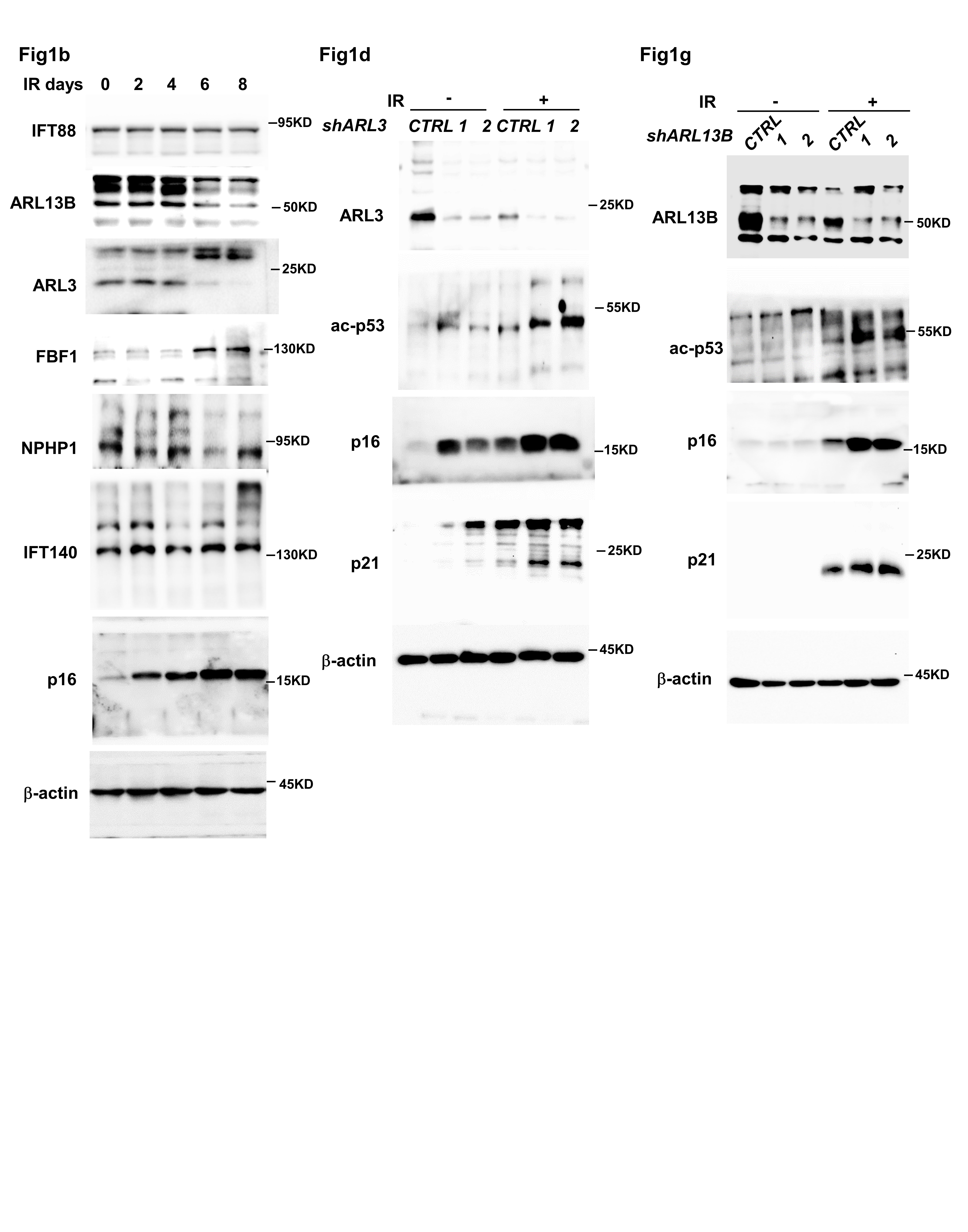


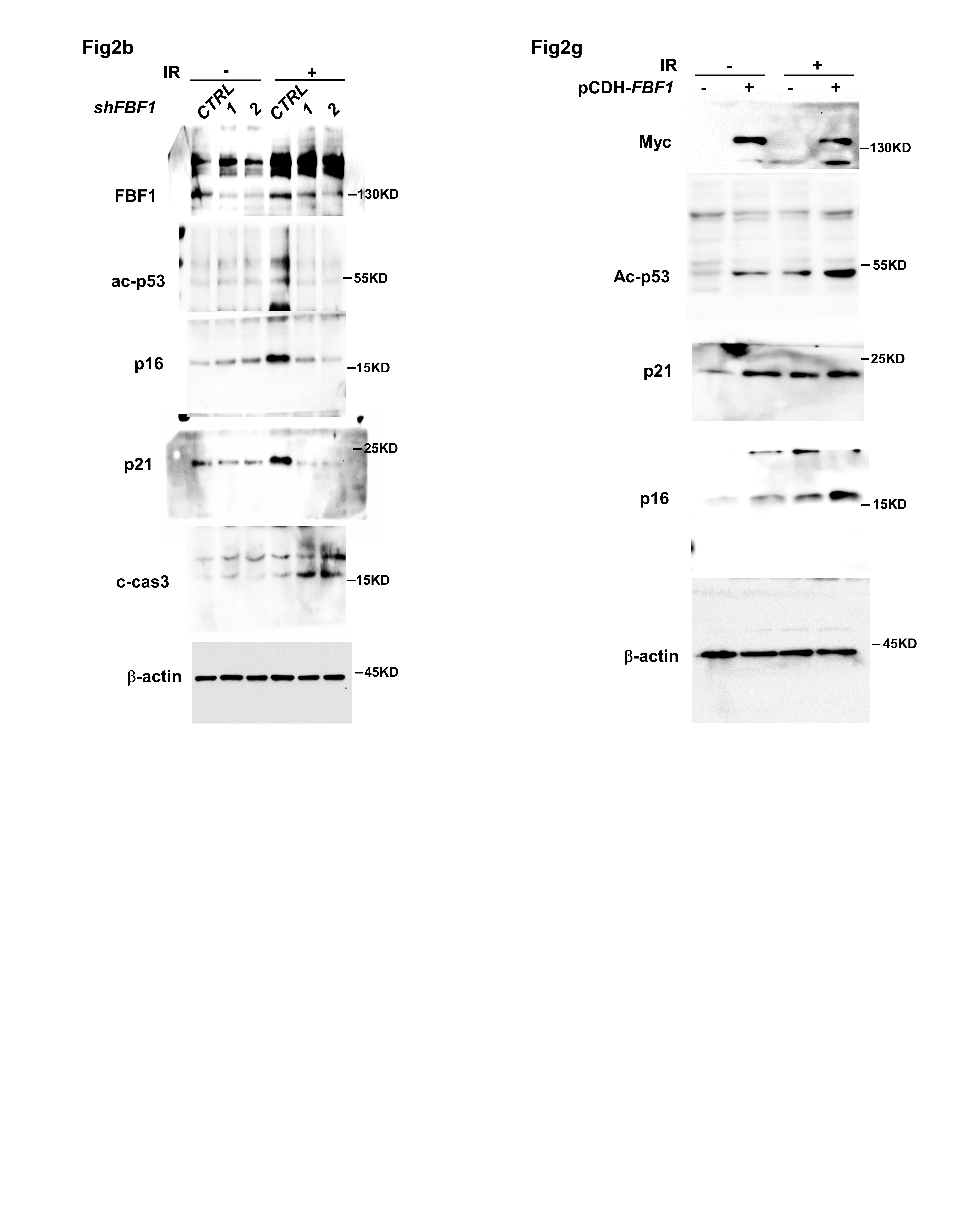


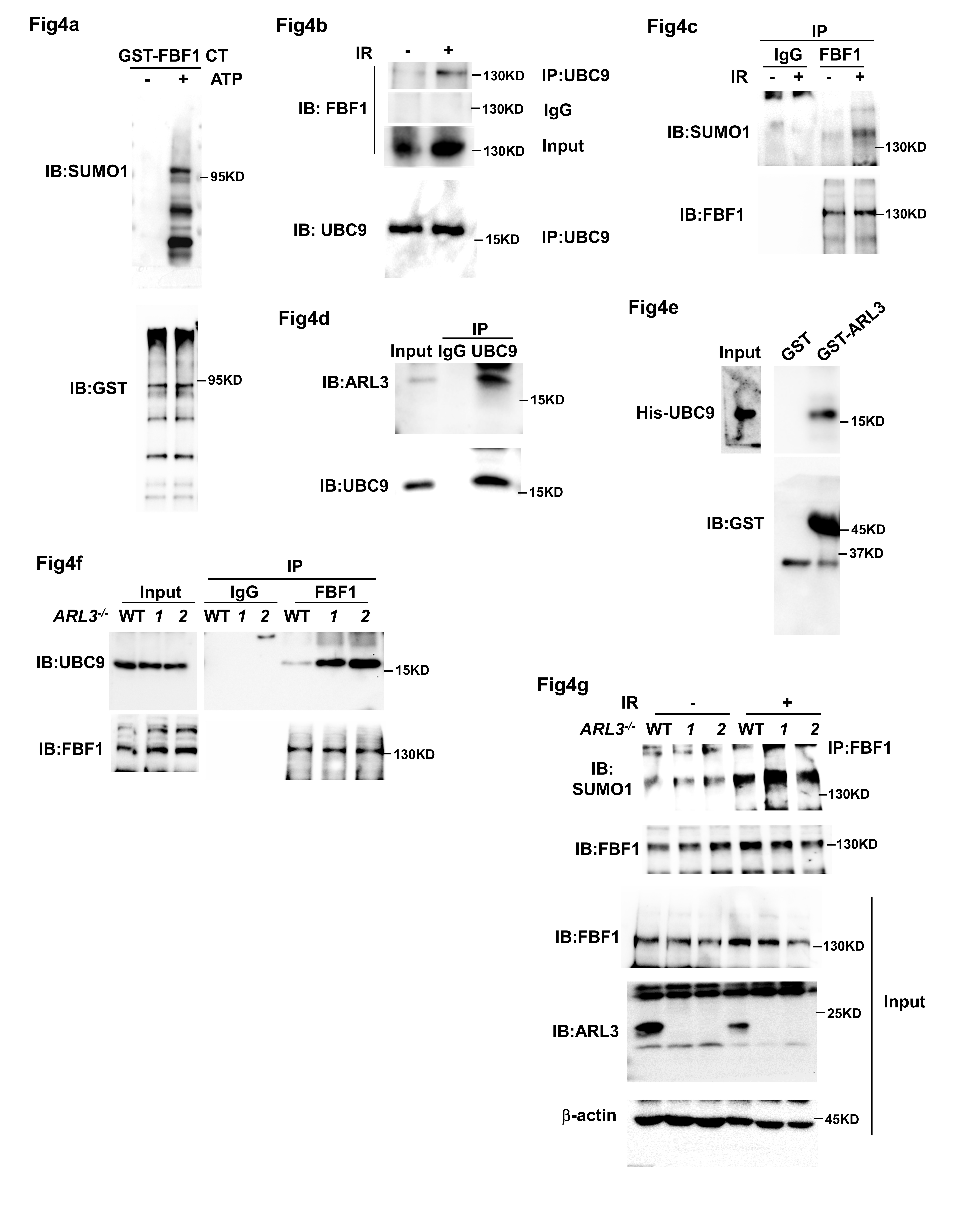


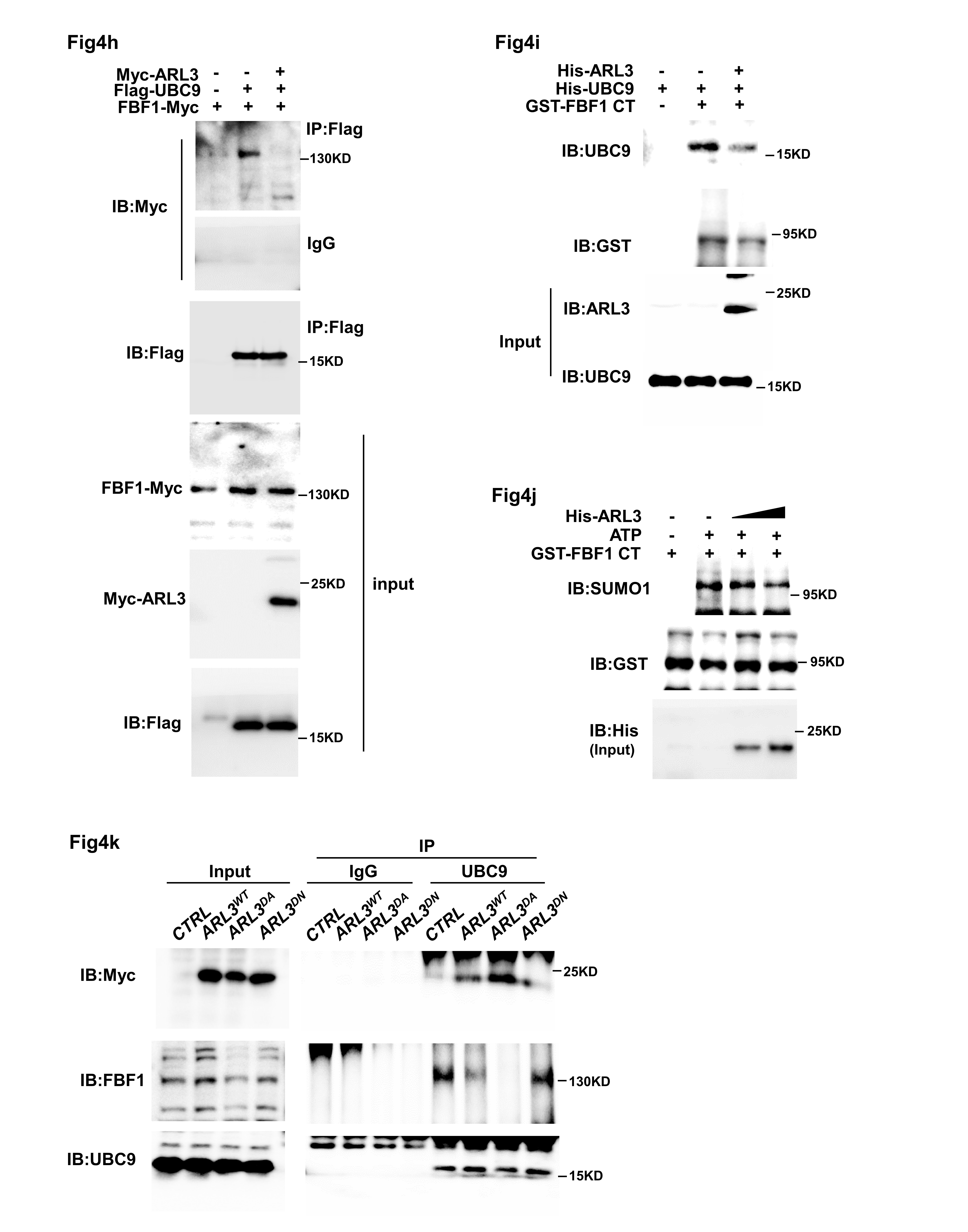


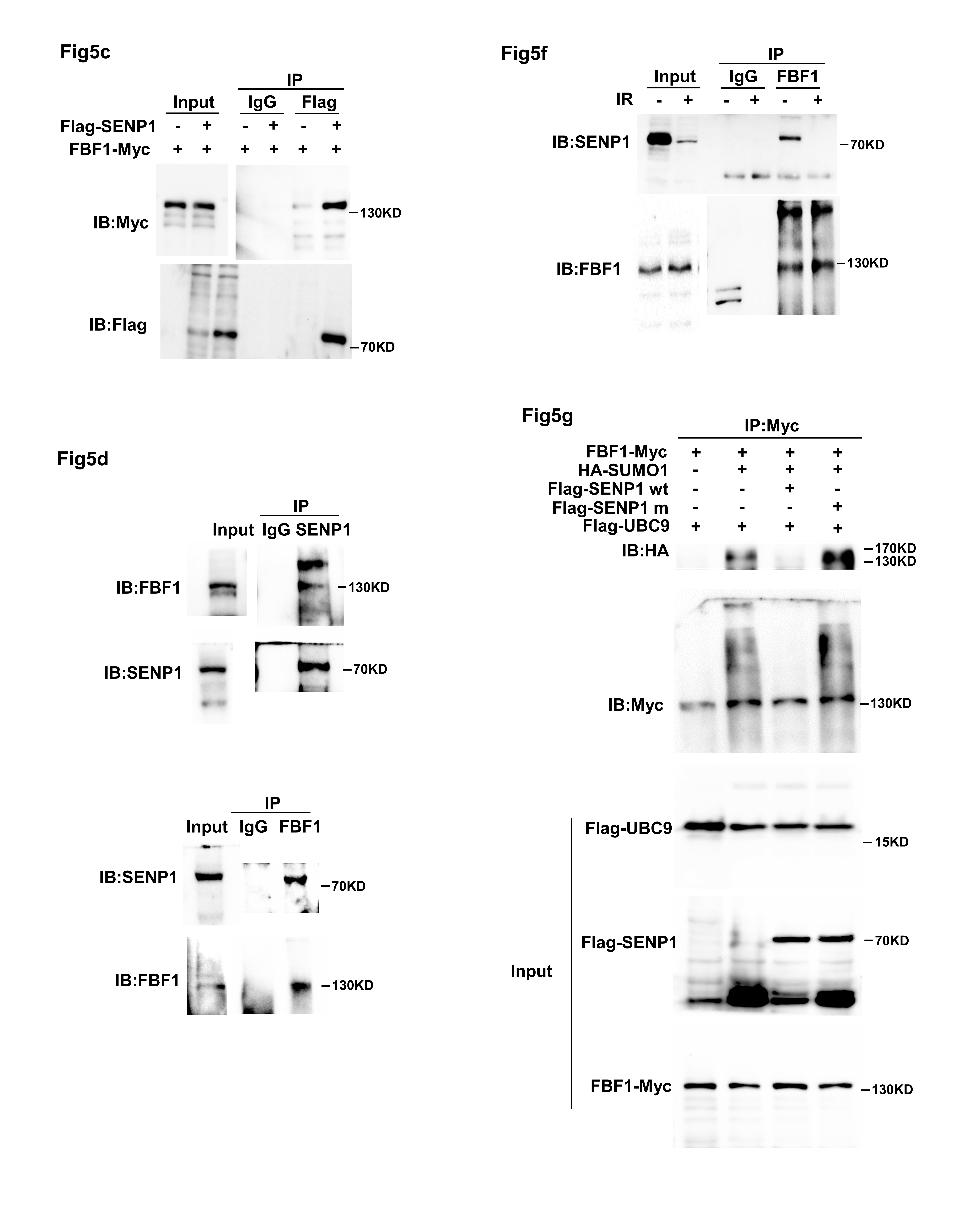


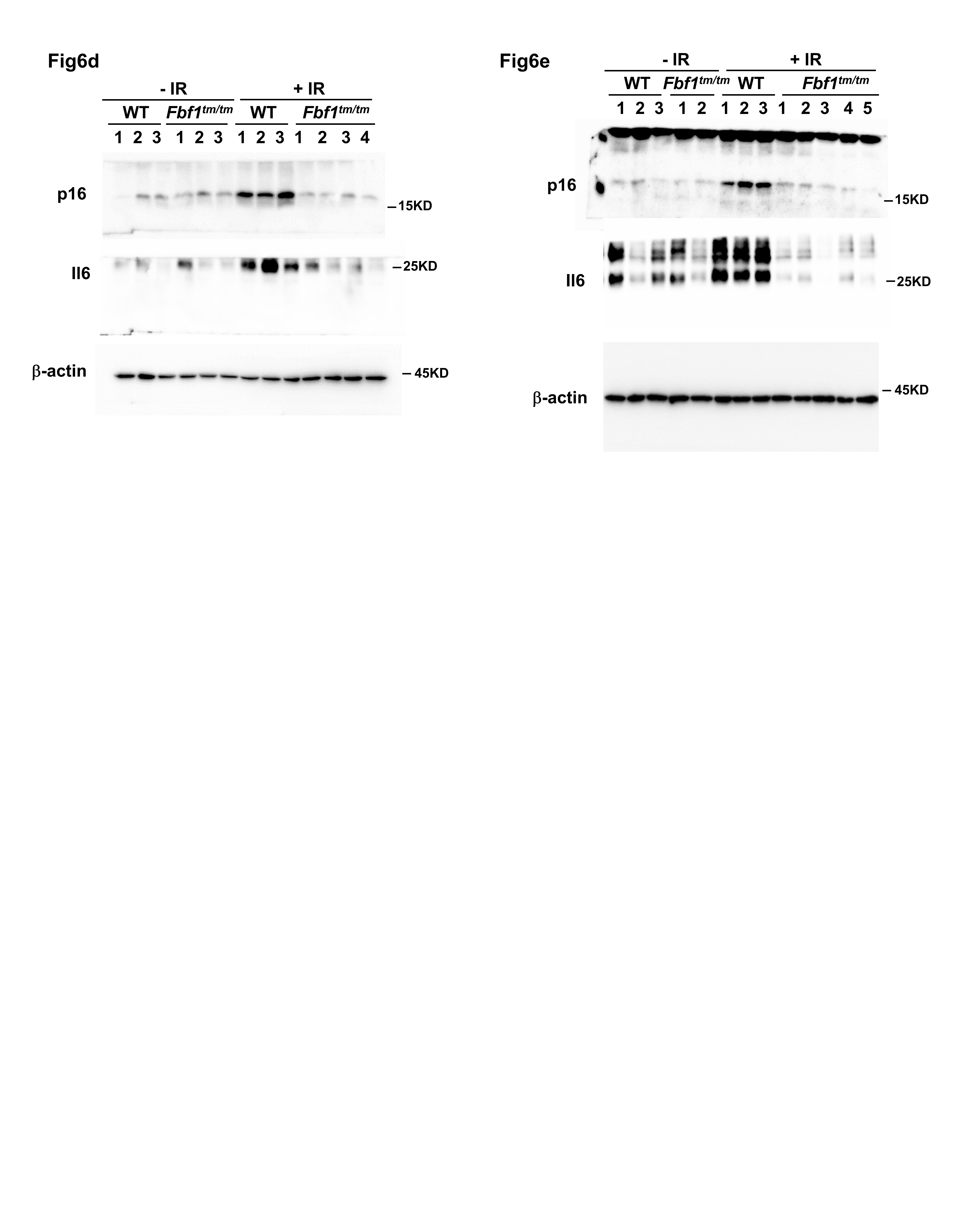


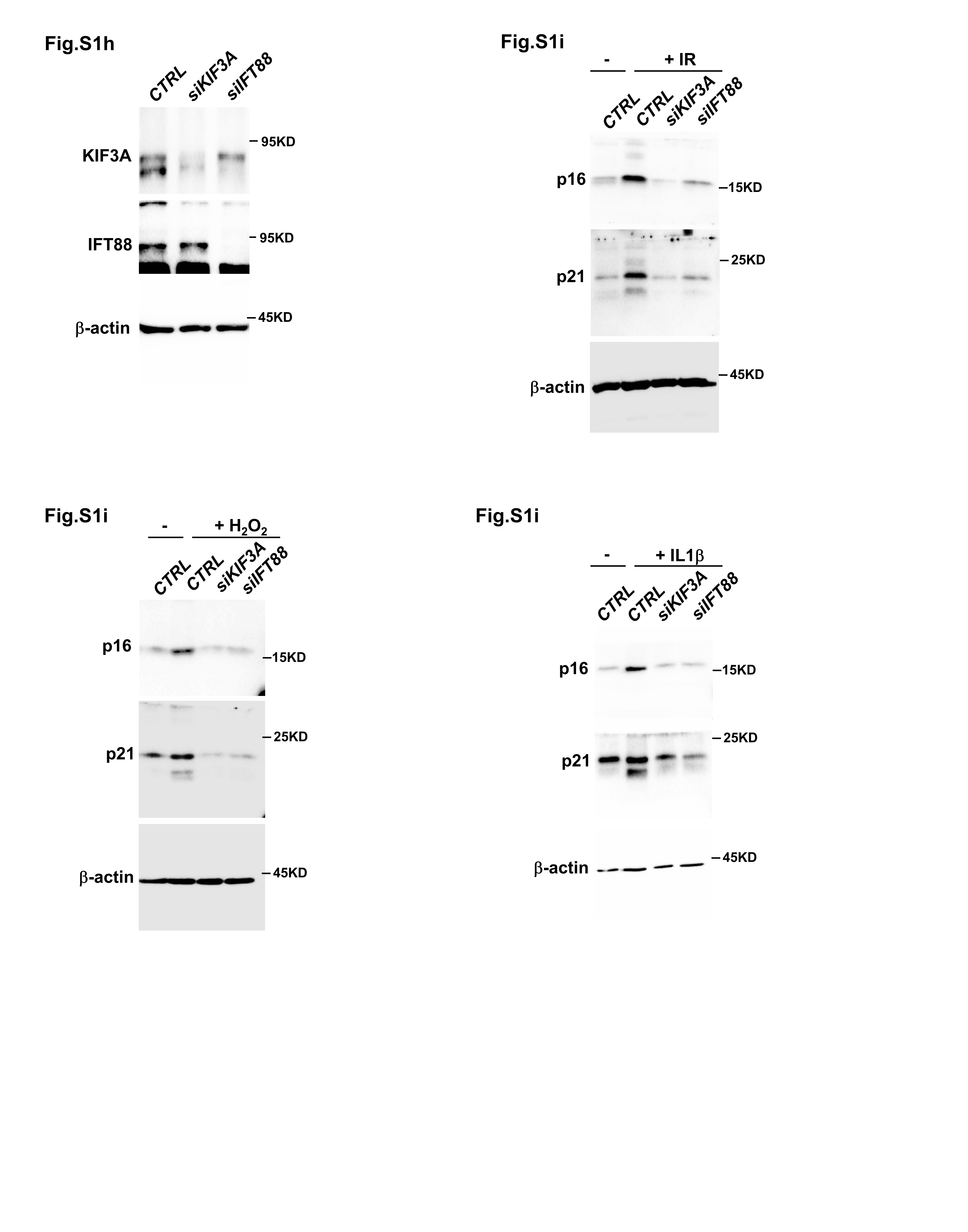


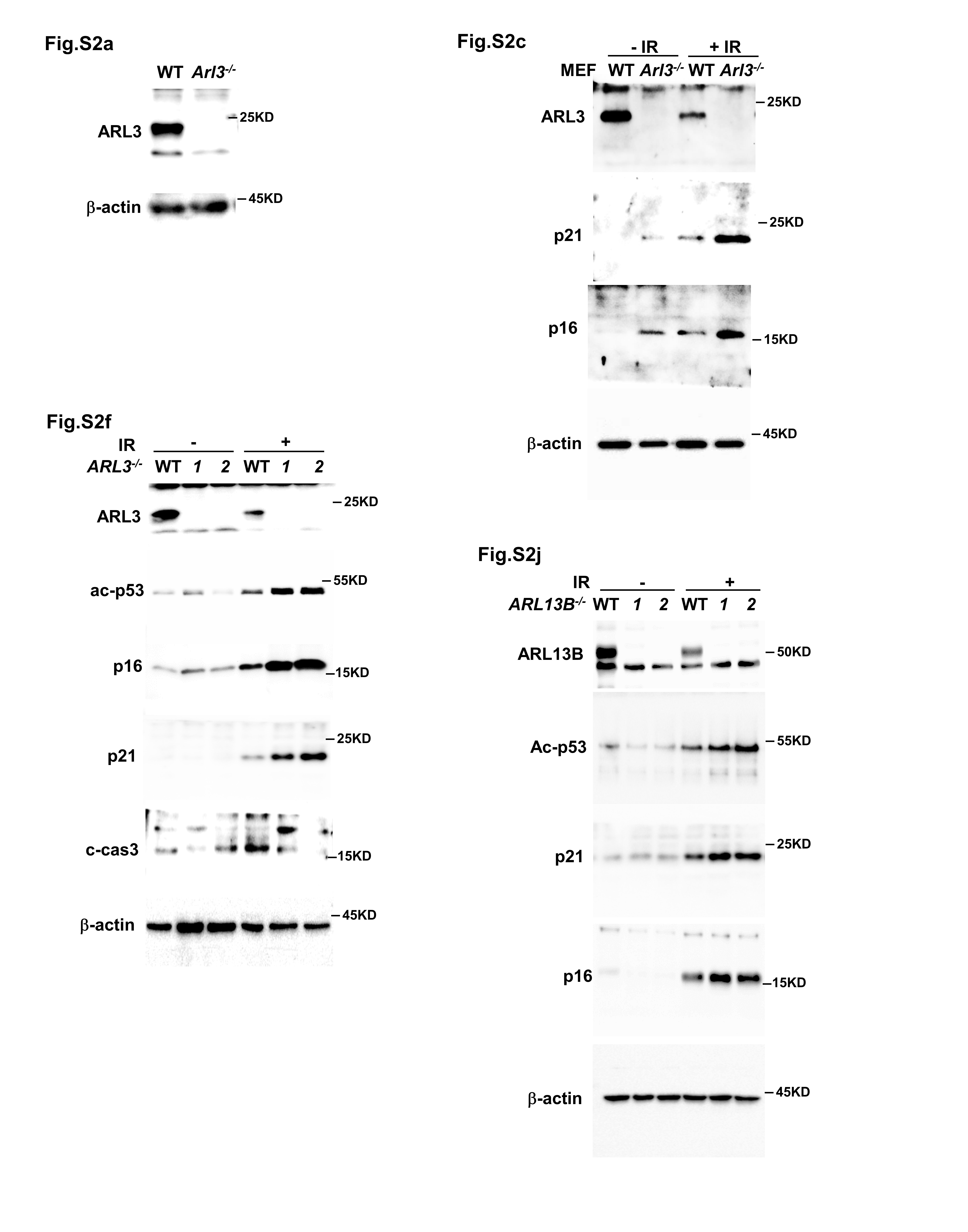


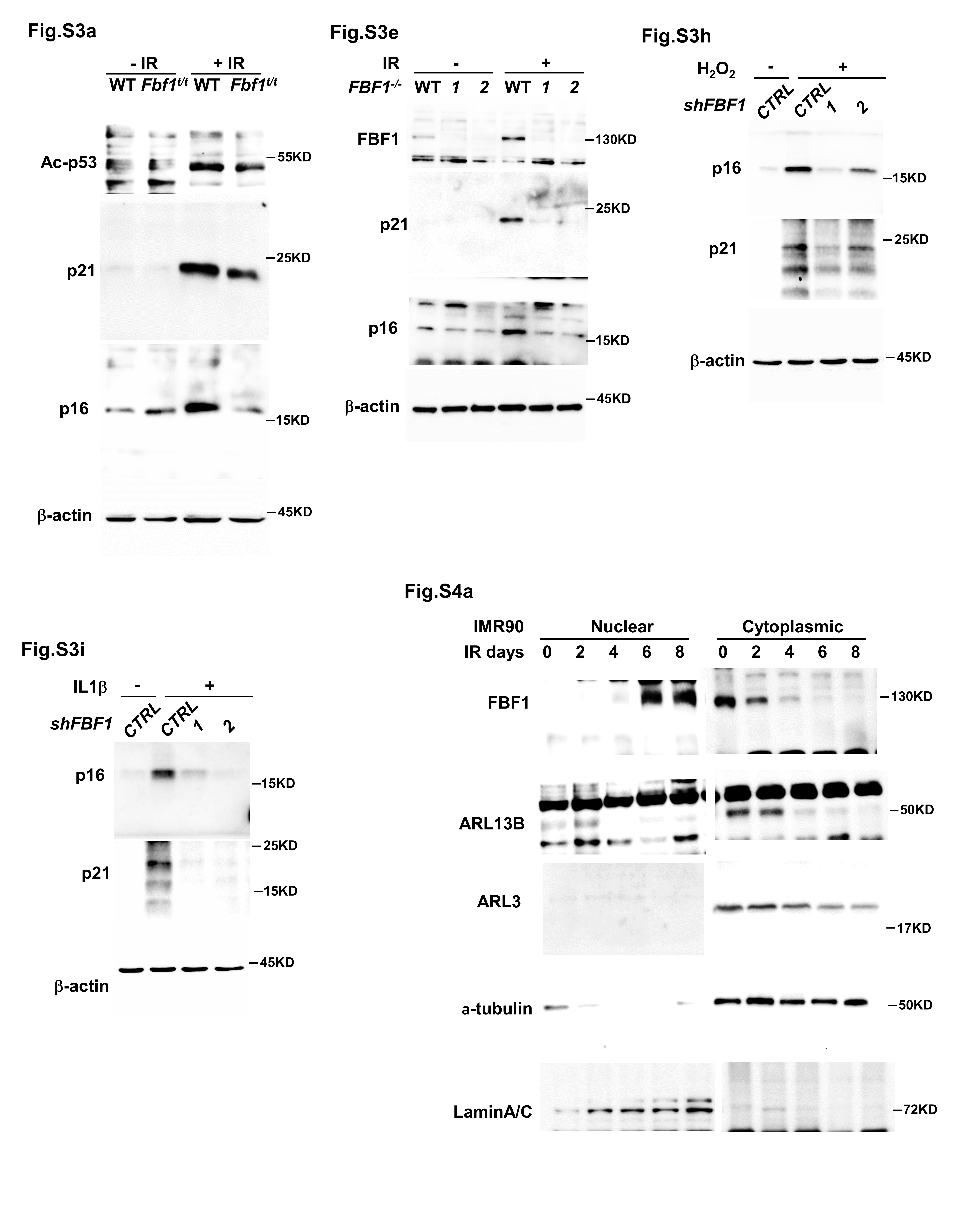


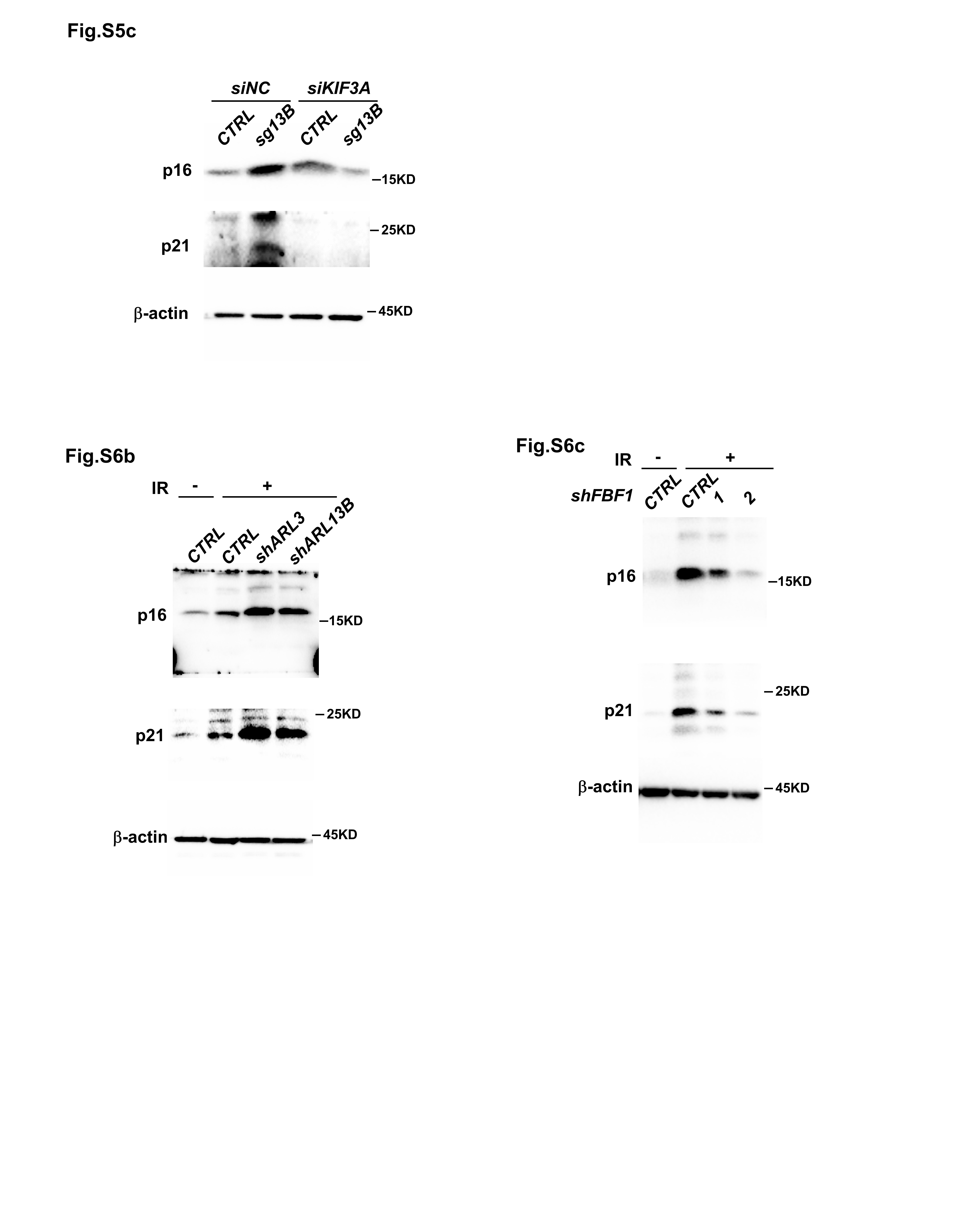


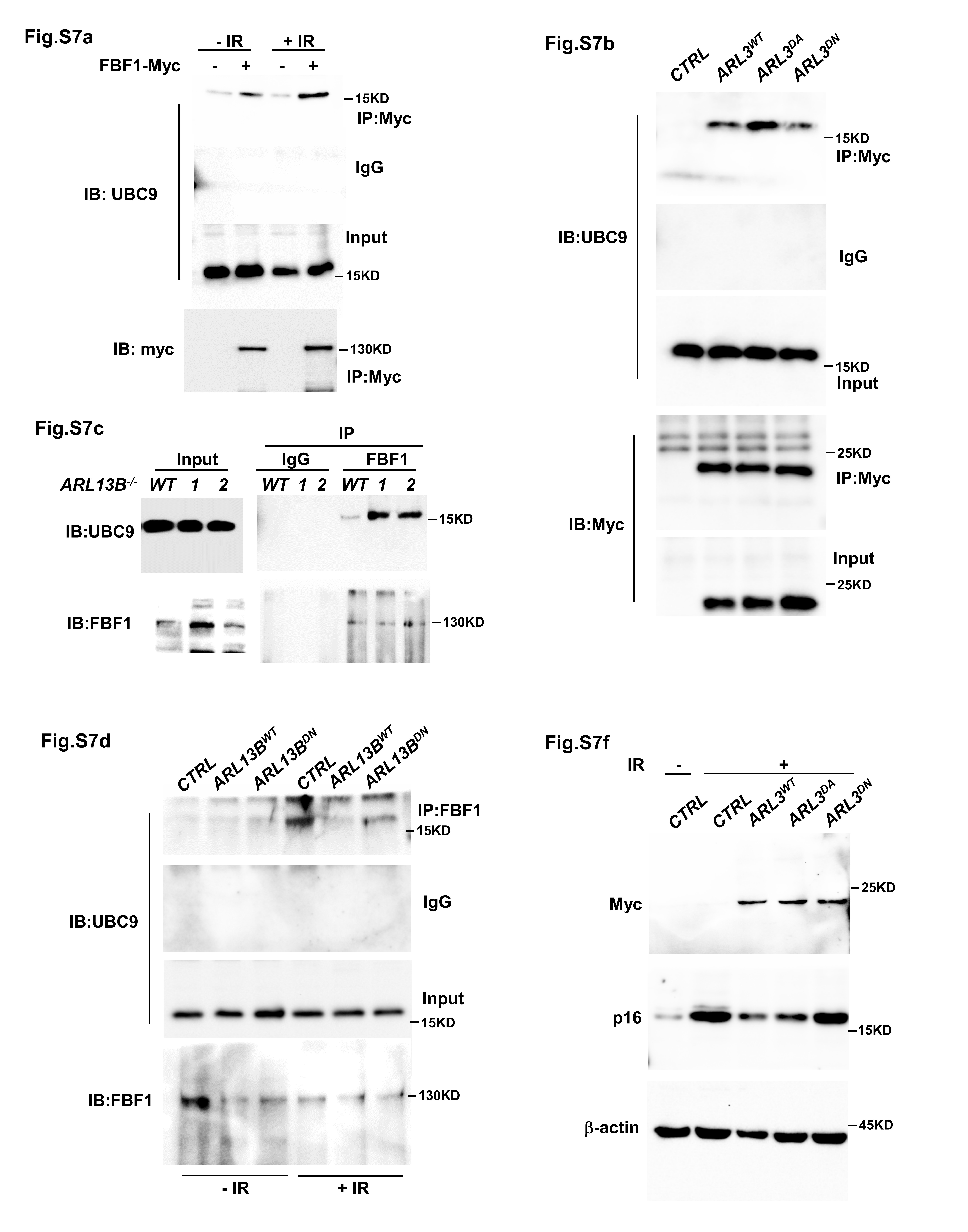


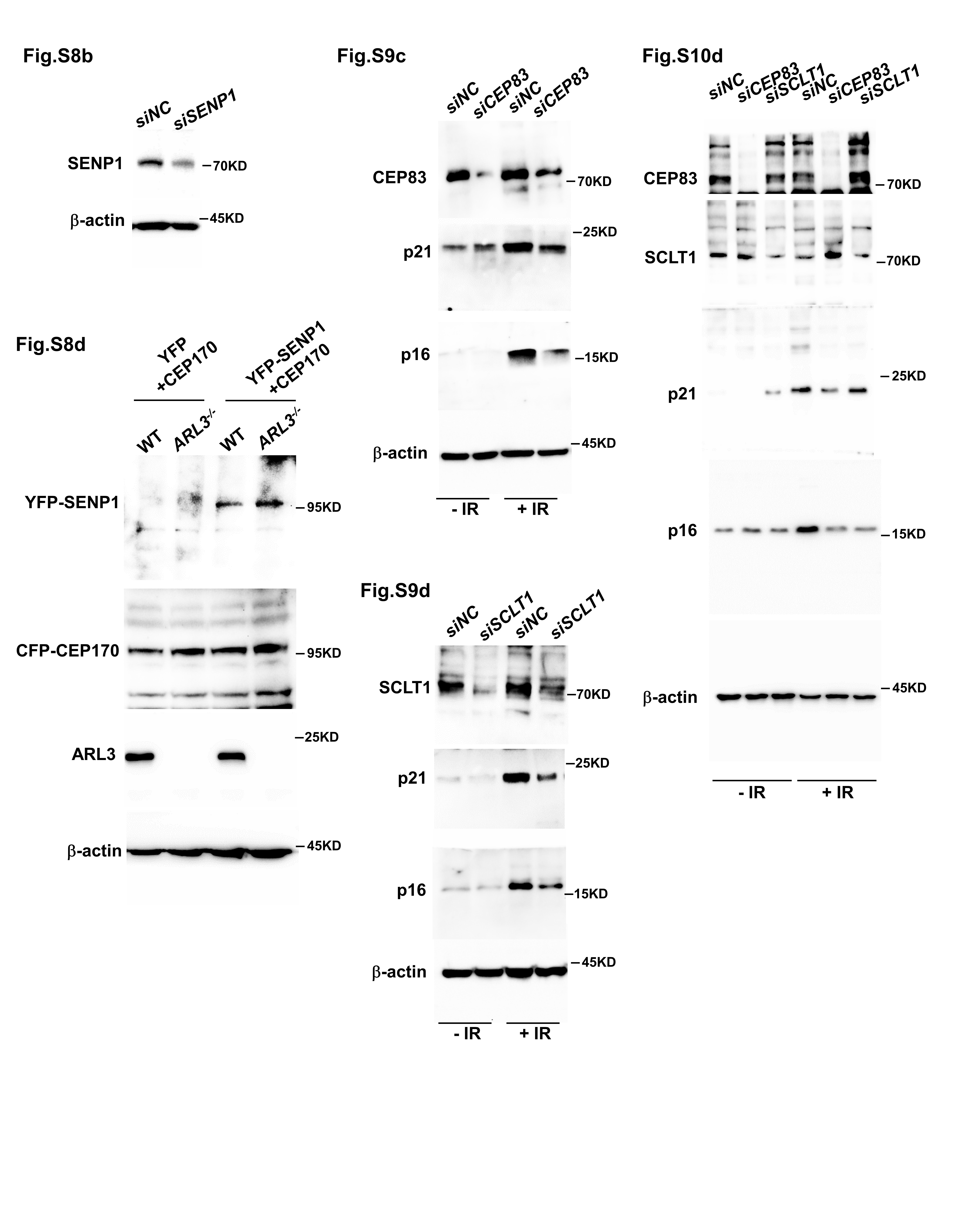


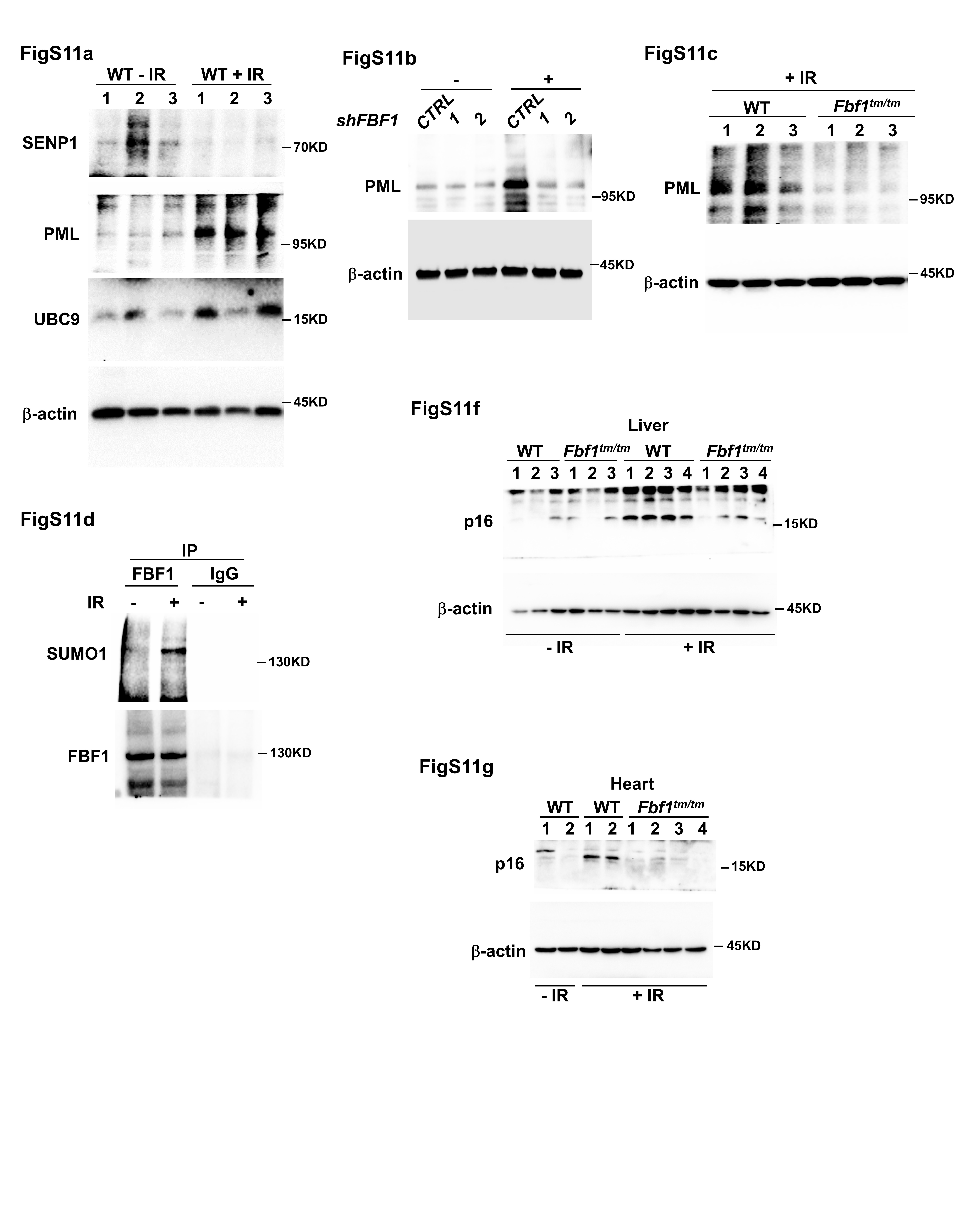

Supplement: Supplementary file 6 — Source Data [file 41467_2023_37362_MOESM6_ESM.zip › Source data file uncropped blots.docx]
